# Metabox: A Toolbox for Metabolomic Data Analysis, Interpretation and Integrative Exploration

**DOI:** 10.1371/journal.pone.0171046

**Published:** 2017-01-31

**Authors:** Kwanjeera Wanichthanarak, Sili Fan, Dmitry Grapov, Dinesh Kumar Barupal, Oliver Fiehn

**Affiliations:** 1 West Coast Metabolomics Center, Genome Center, University of California Davis, Davis, California, United States of America; 2 Biochemistry Department, King Abdulaziz University, Jeddah, Saudi Arabia; Steno Diabetes Center, DENMARK

## Abstract

Similar to genomic and proteomic platforms, metabolomic data acquisition and analysis is becoming a routine approach for investigating biological systems. However, computational approaches for metabolomic data analysis and integration are still maturing. Metabox is a bioinformatics toolbox for deep phenotyping analytics that combines data processing, statistical analysis, functional analysis and integrative exploration of metabolomic data within proteomic and transcriptomic contexts. With the number of options provided in each analysis module, it also supports data analysis of other ‘omic’ families. The toolbox is an R-based web application, and it is freely available at http://kwanjeeraw.github.io/metabox/ under the GPL-3 license.

## Introduction

**Fig 1:**
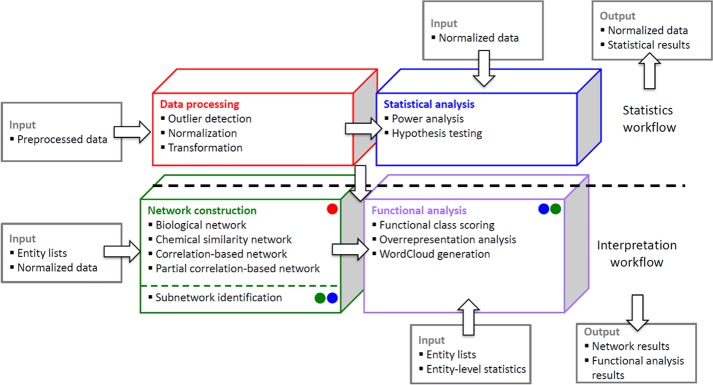
Analysis workflows. Metabox supports in-depth analysis of metabolomic data by including four analysis modules: data normalization (red), statistical analysis (blue), network construction (green) and functional analysis (purple). Outputs from each module are in red, blue, green and purple circles respectively. The tool accepts external inputs on each analysis level. Within metabox, the output from an analysis module can be used for subsequent analyses in the other modules denoted as a colored circle inside a box.

Advances in high-dimensional ‘omic’ platforms have enabled large-scale characterization of molecular phenotypes for a variety of biological systems. Processing, integrating and visualizing modern biological data sets is a formidable task that requires a flexible computational framework to enable a growing variety of genomic, biochemical and phenotypic data types. Key metabolomic data analyses are comprised of four major steps (1) raw data pre-processing including compound identification (2) data processing including data transformation and data normalization (3) statistical analysis and (4) data interpretation. Raw data pre-processing composes of several steps to preprocess raw signals from analytical techniques (e.g. mass spectrometry (MS) and nuclear magnetic resonance (NMR)), which includes noise reduction, peak picking and compound identification [1]. Metabox is independent of tools that were used for raw data preprocessing. Metabox starts with step (2), data processing. Data transformation is defined as the process of converting data into more useful forms of the same data either by mathematical operations (e.g. log-transformation) or by changing formats (e.g. rounding data, or converting units). Data normalization and data transformation are performed on the matrix of imported metabolomic result data to minimize systematic and technical variations before statistical evaluation [1] Subsequently, statistical analyses are used to pinpoint metabolites that are altered based on the experimental design group that therefore must be detailed in the imported data. Statistcal result outputs are then interrogated by downstream modules including network- and pathway-based data analyses and visualization tools [2]. Fig 1 illustrates key analysis steps that can be performed in metabox.

Given the diverse areas of metabolomic data analysis, computational platforms are needed for average users to increase efficiency and to reduce hurdles for in-depth interpretations of metabolomic data along with other ‘omic’ data types. MetaboAnalyst is an online web application that covers comprehensive analysis of metabolomic data [3–5]. The functional module for the integrative analysis is based on metabolomics pathway analysis (MetPA). In difference to MetaboAnalyst, metabox uses a graph database as underlying resource. Pathway relationships in MetaBox are dynamically constructed and therefore, pathway mapping is not limited to static (pre-defined) metabolic pathways as in MetaboAnalyst. [6]. Similarly, a tool like IMPaLa addresses the integration of transcriptomic and metabolomic data on the metabolic pathway contexts but it lacks of an integrative visualization option [7]. In fact, metabolite profiles incorporate environmental and genetic factors into biochemical processes [8]. Combining metabolic information with biological mechanisms of genes and proteins reflects the flow of biological information between layers of cellular regulation. An integrative exploration of metabolomics with secondary lines of molecular ‘omic’ information enables generating new hypotheses such as identification of disease factors [9]. Further tools exist that also exploit the framework of biological networks for multi-omic data exploration, such as MetScape [10], Grinn [2], ConsensusPathDB [11] or 3Omics [12]. Similar to metabox, these tools facilitate the joint visualization of genes, proteins and metabolites in different combinations of biological networks. However, in general these programs are less powerful in data processing and statistical analysis tools. In addition, both MetScape and Grinn lack modules for pathway analyses. All these programs serve the community as open-access software; commercial software such as MetaCore was not available to us for comparison.

We here introduce the R-based web application ‘metabox’ that combines state-of-the-art methods essential for metabolomic data processing, statistical analyses, network-based visualization and functional analyses. The current version of metabox highlights deep analyses of metabolomic data and integrative exploration of metabolites, proteins and genes of interest within several contexts of biological networks such as metabolic pathways, gene regulation and molecular interaction. A number of options are provided, which can be selected to apply on the other omic studies (e.g. transcriptomics and proteomics). Briefly, the following features are integrated in metabox: (i) data normalization and data transformation, (ii) univariate statistical analyses with hypothesis testing procedures that are automatically selected based on users’ study designs, (iii) joint visualization of genes, proteins and metabolites in different combinations of biochemical networks, (iv) calculation of data-driven networks using correlation-based approaches, (v) estimating chemical structure similarity networks from substructure fingerprints, (vi) functional interpretation with overrepresentation analysis, functional class scoring and WordCloud generation and (vii) interactive visualization of information-rich tables and networks. In addition, metabox is distributed as a standard R package which allows ease of distribution, installation and makes internal metabox functions available to be used in custom workflows by advanced users.

## Materials and Methods

Metabox is an R-based software package that is developed as a web application for interactive scientific computing and visualization. The tool is composed of a pre-compiled graph database and R functions essential for the analysis of metabolomic data. The following sections explain each of the tool components in details.

### Internal graph database

The internal graph database is a pre-compiled graph database, which is used for collecting prior knowledge from several resources (S1 Table). The database is required for biological network query functions explained in the following section. A Neo4j graph database (http://neo4j.com/) is used for storing information of molecular entities and their relationships. In this version, the graph database is available for *Homo sapiens*. Upon installation, it will automatically access this pre-compiled human database on our server.

The database schema was adapted from the Grinn package [2] (S1 Fig). In particular, the database includes the majority of biological relationships among molecular entities such as substrate-product relationships (BIOCHEMICAL_REACTION), protein-compound catalysis (CATALYSIS), protein-protein interactions (MOLECULAR_BINDING), gene-encoding proteins (CONVERSION), protein-gene transcription regulations (CONTROL), gene-gene associations (GENETIC_ASSOCIATION), microRNA-gene regulations (CONTROL) and pathway-entity annotations (ANNOTATION) (S2 Table).

Two identifier systems are used in the database: Neo4j internal identifiers (NIDs) and Grinn identifiers (GIDs). The NID is a numeric number generated automatically by the Neo4j database system. The GID uses the authentic identifiers of different domain databases. In particular, GIDs uses ENSEMBL identifiers for genes, miRTarBase nomenclature for micro-RNAs, UniProt entries for proteins, and PubChem CIDs or KEGG numbers for compounds and pathways. User inputs require either GID identifiers or convert other identifiers into NIDs via a converter tool provided in metabox (S3 File).

### R-based functions for analysis workflows

In metabox, metabolomics analysis workflows are divided into statistics and interpretation (Fig 1). The statistics package includes data processing and statistical analysis, while the interpretation workflow is used for biological interpretations of the statistical outputs. Backend functions for both workflows were developed in the R programming language (https://www.r-project.org/).

#### Data processing and statistical analysis

Data processing and statistical analysis are the key tasks of metabolomic data analysis. The aim of data processing (transformation and normalization) is to improve normality of data sets in order to improve comparability of metabolite intensities. Subsequently, statistical tools are used to find significant molecular entities, both for hypothesis testing (univariate analysis) and cluster analysis (multivariate analysis which may serve for hypothesis generation).

- *Data normalization*. Two types of normalization methods are currently implemented: feature-based normalization and sample-based normalization. Both normalizations can be performed sequentially. Sample-based normalization aims to normalize each sample to control for the systematic and technical variance between samples. Several commonly used normalization methods are included in metabox: normalization to sample-specific metadata (such as dry mass), normalization to the sum of all identified metabolites [13], normalization to batch-median values of specific samples data, or LOESS (locally estimated scatterplot smoothing) normalization to specifically introduced quality control or pool samples [14]. LOESS normalizations are performed with span parameters that are automatically selected by cross-validation and batch effect correction [15]. Conversely, metabolite-based normalization aims to make the measured variables more comparable to each other with respect to total variance. Three methods are available in metabox including auto-scaling, Pareto scaling and range scaling [16]. These normalization methods are usually performed for multivariate analyses tools in order to ensure that data heteroscedasticity is reduced to a minimum.
- *Data transformation*. Metabox offers common variable transformation methods including logarithm and power. These methods can be used to improve data normality assumptions for parametric statistical hypothesis testing procedures which can otherwise be sensitive to non-normal distributions, outliers and lack of homogeneity of variances.
- *Exploratory data analysis*. Metabox uses principal components analysis (PCA) score plots for real-time visualization during data processing procedures [16]. It allows users to detect outliers and choose appropriate methods for data normalization and transformation according to the properties of the data structure. In addition, users may select scatters on the PCA score plots and get the corresponding sample information from a donut chart. This helps users to discover unexpected features within the data structure.
- *Univariate Analysis*. Metabox collects a variety of well-established statistical hypothesis testing methods and post hoc analysis procedures (Table 1). In addition, metabox includes corresponding non-parametric testing procedures, post hoc analysis with false discovery rate (FDR) correction on both main effect level and simple main effect level, and power analyses at the entity-level. Bootstrapping is provided as an optional non-parametric testing procedure. Furthermore, metabox automatically and appropriately suggests statistical analysis methods according to the user-input study design. This feature aims to aid users through the depths of statistical terminology.
- *Power analysis* is provided at the entity-level. It covers power analyses for hypothesis testing procedures listed in Table 1. Metabox offers two levels of power analyses: prior to estimate sample size and post-hoc to estimate statistical power.

**Table 1:** List of statistical analysis procedures in metabox.

Study Design	Test	Methods	Following Procedures
Two-independent group	Statistically significant difference exists between means of two independent groups	Welch's t test, Mann-Whitney U test*, t test;	P-values adjusted for FDR correction using Bonjamini-Hochberg procedure [17]
Two-paired group	Mean difference between paired observations is statistically significant different from zero	Welch's t test on difference, Mann-Whitney U test on difference*, Welch's t test on difference;	P-values adjusted for FDR using Bonjamini-Hochberg procedure
Multiple- independent group	There are statistically significant differences between the means of three or more independent groups	Welch ANOVA; Kruskal-Wallis rank sum test*; ANOVA;	Parametric test followed by Games-Howell (or Tukey) post hoc test to compare all possible combinations of group differences; non-parametric test followed by pairwise comparisons using Dunn's procedure [18] with Bonferroni adjustment.
Multiple-paired group	There are statistically significant differences between the means of three or more levels of a within-subjects factor.	Repeated ANOVA; Friedman test*;	Parametric test result corrected by Greenhouse-Geisser procedure [19] for violation of sphericity and followed by Bonferroni post hoc test as suggested by Maxwell and Delaney [20]; non-parametric followed by pairwise comparisons using Wilcoxon signed-rank tests [21] were performed with Bonferroni correction
Two-way independent groups	There is a statistically significant interaction effect between two ways of independent groups	Two-way ANOVA, two-way ANOVA with robust estimation	Results followed by post hoc analysis on main effect level and simple main effect level corresponding to secondary study design structure
Two-way paired groups	There is a statistically significant interaction effect between two within-subject ways	Two-way repreated ANOVA	Results followed by post hoc analysis on main effect level and simple main effect level corresponding to secondary study design structure
Two-way mixed groups	There are differences between independent groups over time	Mixed ANOVA	Results followed by post hoc analysis on main effect level and simple main effect level corresponding to secondary study design structure

* Non-parametric test.

Underlined method denotes the default method in metabox

#### Network construction

Network-based analysis is a promising approach to explore molecular interactions. In addition, networks can be used as a scaffold for mapping experimental results to identify potential markers, altered pathways or active subgraphs. Metabox supports both construction of networks from domain knowledge relationships and from empirical relationships. There are four main functions to construct networks from different contexts.

- *Biological networks* are queried from our pre-compiled graph database that contains *a priori* relationships from several resources in SimpleNetwork and HeterogeneousNetwork analyses. SimpleNetwork analysis is used to query biological networks of one type of relationship (e.g. protein-protein interaction networks) whereas HeterogeneousNetwork analyses query biological networks containing one or combinations of relationship types (e.g. biochemical reaction networks containing substrate-product and compound-enzyme associations). Lists of entities and relationship patterns are illustrated in the database schema (S1 Fig).
- *Weighted correlation networks* are computed from experimental data using pairwise or partial correlation approaches. Pairwise correlation analysis computes associations between every pair of entities. Metabox includes Pearson, Spearman or Kendall correlation analyses based on the WGCNA package [22]. Partial correlation analysis is performed based on the qpgraph package that estimates associations between entities while controlling effects from other entities [23, 24].
- *Chemical structure similarity networks* are computed from PubChem substructure fingerprints by using Tanimoto chemical similarity matrix calculations [25, 26].
- *A high-scoring subnetwork* is enumerated from biological networks, weighted correlation networks or chemical structure similarity networks. This subnetwork identification is based on the BioNet package that calculates scores of network nodes from p-value results of statistical analyses and follows by a heuristic search for identification of the high-scoring subnetwork [27, 28].

#### Functional analysis

Functional analysis is provided to aid biological interpretations of the results from statistical analysis, the network outputs, and user-uploaded list of molecular entities. The current version of metabox supports KEGG [29] pathway-based analysis for compounds, proteins and genes. Additionally, metabox uses Medical Subject Headings (MeSH) [30] for compound interpretations by querying the ‘chemicals and drugs’ category in the PubChem database [31]. Three different analysis options are included.

- *Functional class scoring [32]* or *set enrichment analysis* assesses the significance of annotation terms from entity-level statistics (e.g. p-values of entities calculated from statistical analysis). Metabox integrates commonly used methods of gene set enrichment analysis from the piano package, including [33], Fisher 's combined probability test [34], Stouffer’s method [35], Reporter features [36, 37], Median and Mean. The Fisher’s method computes each set-level statistics by combining log-transformation of entity-level statistics, while the Stouffer’s method calculates from aggregating Z scores of entity-level p-values. The Reporter method is similar to the Stouffer’s method with an additional procedure of background distribution correction. The Median and Mean method are straightforward (i.e. each set-level statistics is the median and mean of entity-level statistics respectively.). The piano package uses a permutation approach to evaluate the significance of entity set of an annotation term.
- *Overrepresentation analysis* is to identify overrepresented annotation terms of the given list of entities using hypergeometric test. For the networks containing multiple types of nodes, the Fisher’s method is used to combine p-values from hypergeometric test.
- *WordCloud generation* is a simple, graphical presentation of annotation terms where the font size of a word corresponding to number of members. It provides a quick summary of annotation terms of the given list of entities without any statistical test. The function is based on the R package tm [38] and the R package wordcloud (https://cran.r-project.org/web/packages/wordcloud/).

### Graphical user interface

The metabox graphical user interface (GUI) was implemented with HTML, JavaScript and CSS. The backend R functions were deployed on a web browser using the OpenCPU JavaScript library [39], which allows users to execute the R functions on a web browser such as Firefox, Chrome, Internet Explorer and Safari. The GUI is a two-column layout with a side navigation bar containing the list of different functions. On the right side, page contents are changed correspondingly to the selected function. Collapsible tree views of MeSH terms are drawn using the D3.js JavaScript library (https://d3js.org/). Word clouds are plotted using the wordcloud package (https://cran.r-project.org/web/packages/wordcloud/). The Cytoscape.js JavaScript library [40] is used to create interactive networks. Network navigation such as pan, zoom and select can be performed using a mouse or a touchpad. The resulting networks can be downloaded as PNG format and tab-delimited text files which can be used in other software.

## Results and Discussions

In this section, the utility of metabox for metabolomic data analysis is demonstrated using two independent data sets that measured metabolomic and gene expression profiles of lung adenocarcinoma and adjacent non-malignant lung tissues [41, 42]. The example data sets, corresponding tutorials and metabox user manual are provided as supplemental files (S1, S2 and S3 Files, respectively).

### Omic data sets

The metabolomic data contain 39 malignant and adjacent non-malignant lung tissue samples measured by gas chromatography time-of-flight mass spectrometry (GC-TOF-MS) and pre-processed by the BinBase database [43]. 462 compounds were measured, 171 of which were structurally identified and associated by PubChem CID. The data from the BinBase database were uploaded to metabox for log transformation before statistical analysis using paired t-test. After Benjamini and Hochberg FDR (BH-FDR) adjustment at 5% (pFDR < 0.05), there were 131 significant compounds between cancer and control tissues.

Gene expression profiles of 58 lung adenocarcinoma and adjacent non-malignant lung tissues were acquired using Illumina HumanWG-6 v3.0 expression BeadChip platform and downloaded from the Gene Expression Omnibus database [44] as transcriptomic data set GSE32863. Differential gene expression analysis comparing lung adenocarcinoma and adjacent non-malignant lung tissues was performed with GEO2R [44]. P-values were adjusted with Benjamini and Hochberg false discovery rate at 5% (pFDR < 0.05). Differential gene expression analysis comparing tumor and control tissues reported 171 out of 21,204 genes in which pFDR < 0.05 and |log2FC| > 2.

### Using metabox for in-depth analysis of metabolomic data

**Fig 2:**
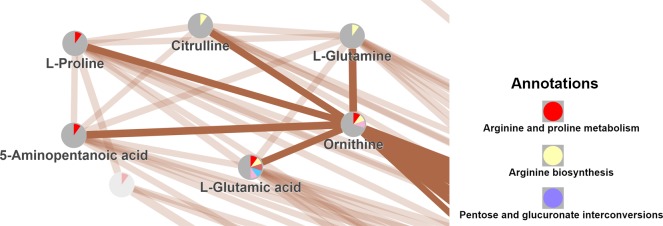
Partial visualization of the entire ‘chemical structure similarity’ network of metabolites in a lung adenocarcinoma study. Chemical similarities between all identified metabolites was calculated from PubChem substructure fingerprints. Network nodes are connected by correlation coefficients using edge thickess for correlations *r*_xy_>0.7. Metabox functional class scoring was applied to estimate significantly enriched pathways (*p*<0.05), yielding arginine/proline metabolism, arginine biosynthesis and pentose/glucuronate interconversions among other pathways. Pathway enrichments are given by color in node pie charts.

The following metabox example shows a comprehensive analysis of metabolomic data, progressing through a variety of common analyses steps including: data processing, statistical analyses, and biochemical display of results within a variety of contexts including structural similarity, pathway and based on other biochemical relationships. The pre-processed metabolic profiles were imported to metabox for data processing and statistical analysis. Subsequently the output was transferred to the network construction part to calculate the chemical structure similarity network of the measured compounds (i.e. 171 compounds have associated PubChem CID). The default threshold was used with a correlation coefficient *r*_xy_> 0.7. The resulting lists of pair-wise metabolic correlations are then displayed as network graph that can be enhanced by further empirical or annotation information. Here, we applied the Functional Class Scoring option to estimate significantly enriched pathways. We found significantly enriched pathways (*p*-value < 0.05) to include arginine and proline metabolism, arginine biosynthesis and pentose and glucuronate interconversions. Fig 2 shows a part of the resulting similarity network overlaid with enriched pathways. Amino acids, ornithine, citrulline and glutamine were metabolically linked through the pathway ‘arginine biosynthesis’. In addition, ornithine was metabolically linked to proline, 5-aminopentanoic acid and glutamic acid through the pathway ‘arginine and proline metabolism’.

### Metabox supports integrative exploration of significant genes and compounds from lung cancer studies in biological network context

Next, we used the lists of significantly dysregulated genes (pFDR < 0.05 and |log2FC| > 2) and significantly different compounds (pFDR < 0.05) from comparisons between paired lung tumors and non-malignant tissues to construct the biological network using the internal graph relationship database. The resulting network was downloaded and enhanced further by mapping with statistical information using Cytoscape [45].

**Fig 3:**
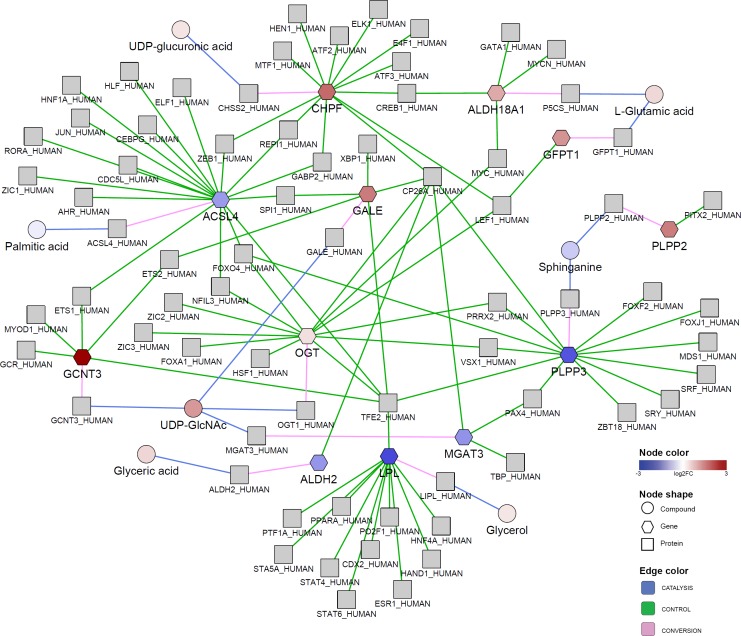
Biological network integrating significant differences in gene and metabolite regulation in lung adenocarcinoma compared to paired control tissues. Significantly different genes and metabolites were mapped onto the metabox internal graph database using enzymes as linking nodes (grey). The resulting network was downloaded and mapped relative changes between tumor and non-tumor tissues using Cytoscape. Graph relationships CONTROL, CONVERSION, and CATALYSIS are labeled by colored edges. The network shows metabolic links between glycerol, palmitic acid, sphinganine, glutamic acid, glyceric acid, UDP-glucuronic acid and UDP-GlcNAc.

The output network shows metabolic links among carbohydrates, glycerol, fatty acids, palmitic acid, sphingolipids, sphinganine, amino acids, glutamic acid and glyceric acid, and glycosylation-related metabolites, including uridine diphosphate glucuronic acid (UDP-glucuronic acid) and uridine diphosphate-N-acetylglucosamine (UDP-GlcNAc) (Fig 3). Combinations of relationships can be explored through such networks, including protein-compound catalysis, protein-encoding genes and protein-gene regulations, integrating information from the regulation of transcription to cellular metabolism. For example, the network outlines metabolic relationships for UDP-GlcNAc and N-acetylglucosamine (GlcNAc) through the corresponding N-acetyltransferase enzymes encoded by *GCNT3* and *OGT* genes, both of which were significantly up-regulated. Modification of nuclear and cytosolic proteins by addition of O-linked β-N-actylglucosamine (O-GlcNAc) at serine or threonine residues or O-GlcNAcylation plays important roles in several cellular processes such as cell signaling, metabolism, transcription regulation and cell division [46]. Evidence indicates that hyper-O-GlcNAcylation is a common feature in cancer [46–48]. UDP-GlcNAc is a crucial GlcNAc donor, which is transferred to protein substrates by O-linked N-acetylglucosamine transferase [46]. Fig 3 also shows that the enzyme-encoding gene *OGT* was linked to protein regulators such as FOXO4 and TFE2 that were connected to genes of phospholipid phosphatase 3 (*PLPP3*) and long-chain-fatty-acid—CoA ligase 4 (*ACSL4*). Both genes were significantly down-regulated in lung cancer compared to control tissues and associated proteins were metabolically linked to significantly decreased compounds sphinganine and palmitic acid respectively. The *OGT* gene was also linked to transcription factors MYC (an oncoprotein found to involve in initiation and maintenance of several cancers [47, 49]) and LEF1, which in turn were associated to genes *ALDH18A1* and *GFPT1*. Both genes were significantly up-regulated in lung cancer compared to control samples and encoded proteins were metabolically linked to significantly increased glutamic acid. In addition, *ALDH18A1* shared the same transcription factor CREB1 with up-regulated gene *CHPF* of glucuronic acid transferase that was connected to substrate UDP-glucuronic acid.

This example demonstrates the use of metabox to integrate information from metabolic and gene expression results for joint visualization in the network context. The resulting network lists and shows relationships among protein regulators, genes, enzymes and metabolites related to amino acid, fatty acid and lipid metabolism, and glycosylation that can be functionally tested in subsequent studies.

### Comparison to existing tools

To date, a variety of bioinformatics tools exist for metabolomic data analysis, however, not all of them support the comprehensive analysis and integrative exploration of metabolomic data. Metabox is a freely available tool that consolidates a number of approaches for data processing, statistical analysis, network construction, integrative visualization and functional interpretations of metabolomic data in one software package. Table 2 provides an overview of tools that contains comparable features to metabox. It also highlights that metabox excels in the scope of statistics and integrative exploration with other ‘omic’ data in network contexts and functional analysis.

**Table 2:** Comparison with existing tools for analysis and interpretation of metabolomic data.

Tools	Metabox	MetaboAnalyst	ConsensusPathDB	MetScape	3Omics	Grinn
**Input**	Gene lists, protein lists, compound lists, omic data, statistical values	Metabolomic data, mass spectral data, Zipped file of NMR data, mass spectral peak lists or mass spectral data	Gene lists, protein lists, compound lists, statistical values	Gene lists, compound lists, correlation values, statistical values	Omic data	Gene lists, protein lists, compound lists, omic data
**Data processing**	Yes	Yes	No	No	No	No
**Statistical analysis**	Univarate and power analysis, automatically suggest analysis method	Univariate and multivariate analysis, clustering, classification	No	No	No	No
**Network construction**	Biological-, weighted correlation- and chemical structure similarity network, subnetwork identification	No	Biological network	Biochemical network	Biochemical and weighted correlation network	Biochemical and weighted correlation network
**Functional class scoring**	Yes	Yes	Yes	No	No	No
**Overrepresentation analysis**	Yes	Yes	Yes	No	Yes	No
**WordCloud generation**	Yes	No	No	No	No	No
**Output**	Tab-delimited files for statistical results, network outputs, and functional analysis result, PNG file for network image, PDF, SVG or PNG file for WordCloud image	CSV files for analysis results, PDF, SVG, PNG or TIFF file for image	Tab-delimited files for network outputs, and functional analysis result	Cytoscape output files	Tab-delimited files for network outputs, and functional analysis result	Tab-delimited files for network outputs
**GUI**	Web-based	Web-based	Web-based	Cytoscape-based	Web-based	Web-based

### Limitations and future directions

The main aim of metabox is to support comprehensive analysis workflows of metabolomics and the integrative exploration with other ‘omic’ data. For the statistics workflow, we included standard normalization, transformation and statistical analysis approaches optimized for metabolomic data. While data processing approaches can be selectively applied on any ‘omic’ data sets, computational time is increased in larger data sets such as transcriptomics and proteomics. Furthermore, when using bootstrapping procedures, run times increase significantly. The current release of metabox does not yet provide a full list of all multivariate or advanced statistics tools (e.g. linear regression and Bayesian models) to support further statistical-based integration of multi-omic data sets. R-packages for these tools exist and will be integrated into the toolbox over time.

In the part of interpretation workflow, metabox supports joint visualization of molecular entities in several combinations of biological networks. The present databases include domain knowledge relationships available only for human from limited resources. We plan to update the current databases using more information from pre-compiled databases such as ConsensusPathDB. In addition, a function to combine different network outputs will also be added. Current functional interpretations are in the contexts of KEGG pathways and MeSH chemicals and drugs categories for compounds. We are investigating to include more types of biological annotations such as disease associations and gene ontology (GO) annotations [50], however, addition of further databases requires careful validation and suitability assessments.

## Conclusions

In this study, we propose an alternative toolbox for thorough analysis and integrative exploration of metabolomic data. Metabox includes widely used statistical methods to process and identify keys entities of input experiments, offers different integrative analysis methodologies and provides interactive visualization to facilitate biological interpretations. The tool is embedded with a graph database and supports both knowledge-based and data-driven network construction. The design of GUI as an intuitive web interface aims to support bench biologists to simply perform data analysis. Metabox is also run as a standard R package for advanced users to use in combination with other R projects. The development of metabox highlights the needs of research communities for the efficient analysis, integration and interpretation of metabolomic studies.

## Supporting Information

S1 Table List of resources and type of information collected in the graph database. (PDF)

S2 Table List of nodes and relationships. (PDF)

S1 Fig Database schema. The database schema illustrates molecular entities and their relationships stored in the graph database. Ovals denote molecular entities and round rectangular indicate relationships.
(TIF)

S1 File Example data sets. (ZIP)

S2 File Tutorials. (PDF)

S3 File User manual. (PDF)
