# Supplementary material for: Metabox: A Toolbox for Metabolomic Data Analysis, Interpretation and Integrative Exploration
Source: PLoS One. 2017 Jan 31;12(1):e0171046. doi: 10.1371/journal.pone.0171046 (PMC5283729; doi:10.1371/journal.pone.0171046)
Supplement: S1 Table — (PDF) [file pone.0171046.s001.pdf]

S1 Table. List of resources and type of information collected in the graph database.

Database	Entity type	Relationship type	Version
PubChem compounds [1]	Compound	-	March 2016 (Chemical and drugs category)
UniProt [2]	Protein	-	March 2016
ENSEMBL [3]	Gene	Conversion	GRCh38.p5
KEGG	Compound, Pathway	Annotation, Biochemical reaction, Catalysis	78.0
miRTarBase	MicroRNA	Control	6.1
BioGRID	-	Genetic association, Molecular binding	3.4.134
Pathway Commons	-	Control	7.0

References

1. Kim S, Thiessen PA, Bolton EE, Chen J, Fu G, Gindulyte A, et al. PubChem Substance and Compound databases. Nucleic acids research. 2016;44(D1):D1202-13. Epub 2015/09/25. doi: 10.1093/nar/gkv951. PubMed PMID: 26400175; PubMed Central PMCID: PMC4702940.
2. UniProt C. UniProt: a hub for protein information. Nucleic acids research. 2015;43(Database issue):D204-12. Epub 2014/10/29. doi: 10.1093/nar/gku989. PubMed PMID: 25348405; PubMed Central PMCID: PMC4384041.
3. Cunningham F, Amode MR, Barrell D, Beal K, Billis K, Brent S, et al. Ensembl 2015. Nucleic acids research. 2015;43(Database issue):D662-9. Epub 2014/10/30. doi: 10.1093/nar/gku1010. PubMed PMID: 25352552; PubMed Central PMCID: PMC4383879.
