# Supplementary material for: Metabox: A Toolbox for Metabolomic Data Analysis, Interpretation and Integrative Exploration
Source: PLoS One. 2017 Jan 31;12(1):e0171046. doi: 10.1371/journal.pone.0171046 (PMC5283729; doi:10.1371/journal.pone.0171046)
Supplement: S2 Table — (PDF) [file pone.0171046.s002.pdf]

S2 Table. List of nodes and relationships.

Nodes	
Compound	Small molecules including all metabolites and drugs
Gene	A region of DNA encoding a functional product that can be inherited through DNA replication
Pathway	A set of interactions forming a network
Protein	A sequence of amino acids
RNA	A sequence of ribonucleotides
**Relationships**	
ANNOTATION	Assigning a defined concept to an entity
BIOCHEMICAL_REACTION	An interaction involving substrates and their products
CATALYSIS	An interaction involving a catalyst and molecular entities of the catalyzed reaction
CONTROL	A controller regulates, modifies, or influences the other (a controlled)
CONVERSION	An entity is transformed to another entity
GENETIC_ASSOCIATION	Considering gene-gene interactions when genetic perturbations of both genes have a combined phenotypic effect
MOLECULAR_BINDING	An interaction involving molecular contact between entities

Adopted from Biological pathways exchange (BioPAX) [1]

References

1. Demir E, Cary MP, Paley S, Fukuda K, Lemer C, Vastrik I, et al. The BioPAX community standard for pathway data sharing. Nature biotechnology. 2010;28(9):935-42. Epub 2010/09/11. doi: 10.1038/nbt.1666. PubMed PMID: 20829833; PubMed Central PMCID: PMC3001121.
