# Supplementary figures and images for: Metabox: A Toolbox for Metabolomic Data Analysis, Interpretation and Integrative Exploration
Source: PLoS One. 2017 Jan 31;12(1):e0171046. doi: 10.1371/journal.pone.0171046 (PMC5283729; doi:10.1371/journal.pone.0171046)

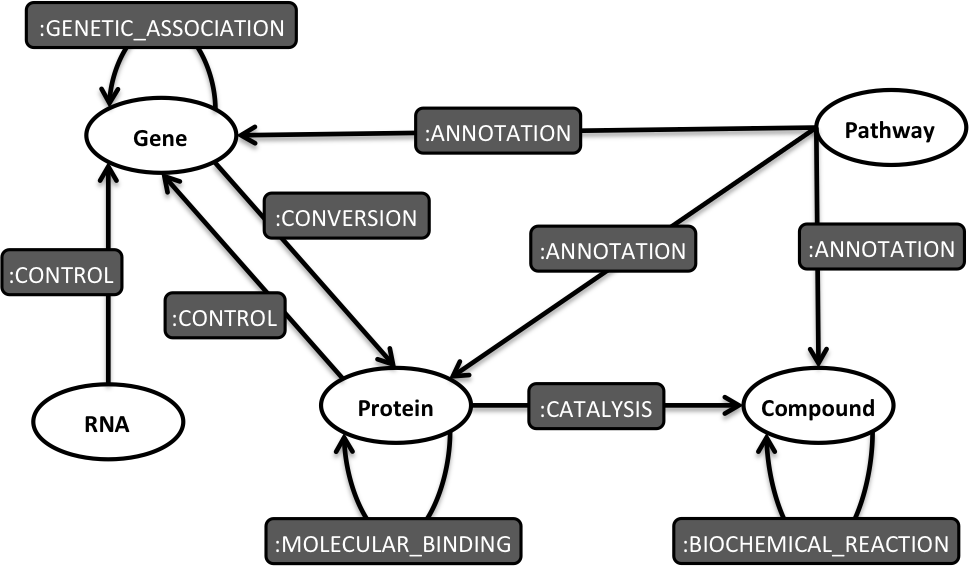

Supplement: S1 Fig — The database schema illustrates molecular entities and their relationships stored in the graph database. Ovals denote molecular entities and round rectangular indicate relationships. (TIF) [file pone.0171046.s003.tif]
