# Supplementary material for: Metabox: A Toolbox for Metabolomic Data Analysis, Interpretation and Integrative Exploration
Source: PLoS One. 2017 Jan 31;12(1):e0171046. doi: 10.1371/journal.pone.0171046 (PMC5283729; doi:10.1371/journal.pone.0171046)
Supplement: S2 File — (PDF) [file pone.0171046.s005.pdf]

metabox: a toolbox for metabolomic data analysis, interpretation and integrative exploration

Kwanjeera Wanichthanarak, Sili Fan, Dmitry Grapov, Dinesh Barupal and Oliver Fiehn

9 January 2017

TUTORIALS

A collection of tutorials for using metabox is provided here to illustrate important features of metabox. These tutorials are corresponding to Results and Discussion section of the main manuscript.

0. Example data sets

The example data sets are based on the published studies about lung adenocarcinoma (transcriptomic data (1) and metabolomic data (2)).

The metabolomic data set containing 39 malignant and adjacent non-malignant lung tissue samples was measured by gas chromatography time-of-flight mass spectrometry (GC-TOF-MS) (2) and pre-processed by the BinBase database (3). 462 compounds were measured, 171 of which have associated PubChem CID. This metabolomic data set was prepared in the input format used in metabox, which is in *metabolomics.xlsx*.

Metabox was used for the statistical analysis comparing tumor and control tissues. For users' convenience, we filtered the list of significantly different compounds comparing tumor and control tissues from the analysis result and provided in *pubchem_stats.txt*.

The transcriptomic data, GEO accession: GSE32863 (1), contain gene expression profiles of 58 lung adenocarcinoma and adjacent non-malignant lung tissues using Illumina HumanWG-6 v3.0 expression BeadChip platform. Differential gene expression analysis comparing lung adenocarcinoma and adjacent non-malignant lung tissues was performed with GEO2R (4). P-values were adjusted with Benjamini and Hochberg false discovery rate at 5% (pFDR < 0.05). Differential gene expression analysis comparing tumor and control tissues reported 171 out of 21,204 genes in which pFDR < 0.05 and |log2FC| > 2. For users' convenience, we filtered the list of significantly different genes comparing tumor and control tissues from the analysis result and provided in *ensembl_stats.txt*.

Table 1. List of example data sets

File	Description	Input of section
metabolomics.xlsx	Metabolic profiles of 39 lung malignant and adjacent non-malignant tissues pre-processed by the BinBase database.	Section 2
pubchem_stats.txt	Multi-column table of significant compounds and associated statistical values computed by metabox.	Section 3
ensembl_stats.txt	Multi-column table of filtered genes and associated statistical values computed by GEO2R.	Section 3

1. Load required libraries and run the metabox using R-terminal

```
## Load library
> library(metabox)
> library(opencpu)

## Run metabox on a web browser
> opencpu$browse(‘library/metabox/www’)
```

2. Using metabox for in-depth analysis of metabolomic data

This tutorial shows that metabox is used for deep analysis (data processing, statistical analysis, metabolic network construction and functional interpretation) of metabolomic data. The example metabolomic data set is in *metabolomics.xlsx*. It is uploaded to metabox for log transformation before statistical analysis using paired t-test (Fig 1). The output from the data normalization and the statistical analysis is transferred to the network construction part to calculate the chemical structure similarity network of the PubChem compounds. The default threshold of correlation coefficient > 0.7 was used. The resulting network is enhanced by further mapping with annotation information. In this case, we applied Functional class scoring option to estimate significantly enriched pathways of network nodes by taking metabolic profiles in to account (see Fig 2 for all the steps).

Alternatively, the output from statistical analysis can be passed to one of the functional analysis method to aid interpretation of measured compounds. In this case, we applied WordCloud option to quickly summarize KEGG pathways of the compounds (see Fig 3 for all the steps). The top ten categories of measured compounds include Central carbon metabolism in cancer, Protein digestion and absorption, Aminoacyl-tRNA biosynthesis, Purine metabolism, Pyrimidine metabolism, Glyoxylate and dicarboxylate metabolism, Arginine and proline metabolism, Pentose and glucuronate interconversions, Galactose metabolism and Fatty acid biosynthesis sorted by the number of compounds in each category. This example shows that after statistical analyses, the result can be interpreted with pathway information.

3. Integrative exploration of significant genes and compounds in biological network context

Metabox is used for joint exploration of the lists of significant genes and compounds from comparisons between tumor and non-tumor tissues in the context of biological networks. The *ensembl_stats.txt* and *pubchem_stats.txt* are used to construct the biological network outlining relationships between genes and compounds as *(:Protein)-[:CONTROL]->(from:Gene)-[:CONVERSION]->(:Protein)-[:CATALYSIS]->(to:Compound)*. The resulting network including all attributes can be downloaded for advance exploration using visualization software such as Cytoscape (5). Fig 4 shows all the steps to construct the network with metabox.

FIGURES

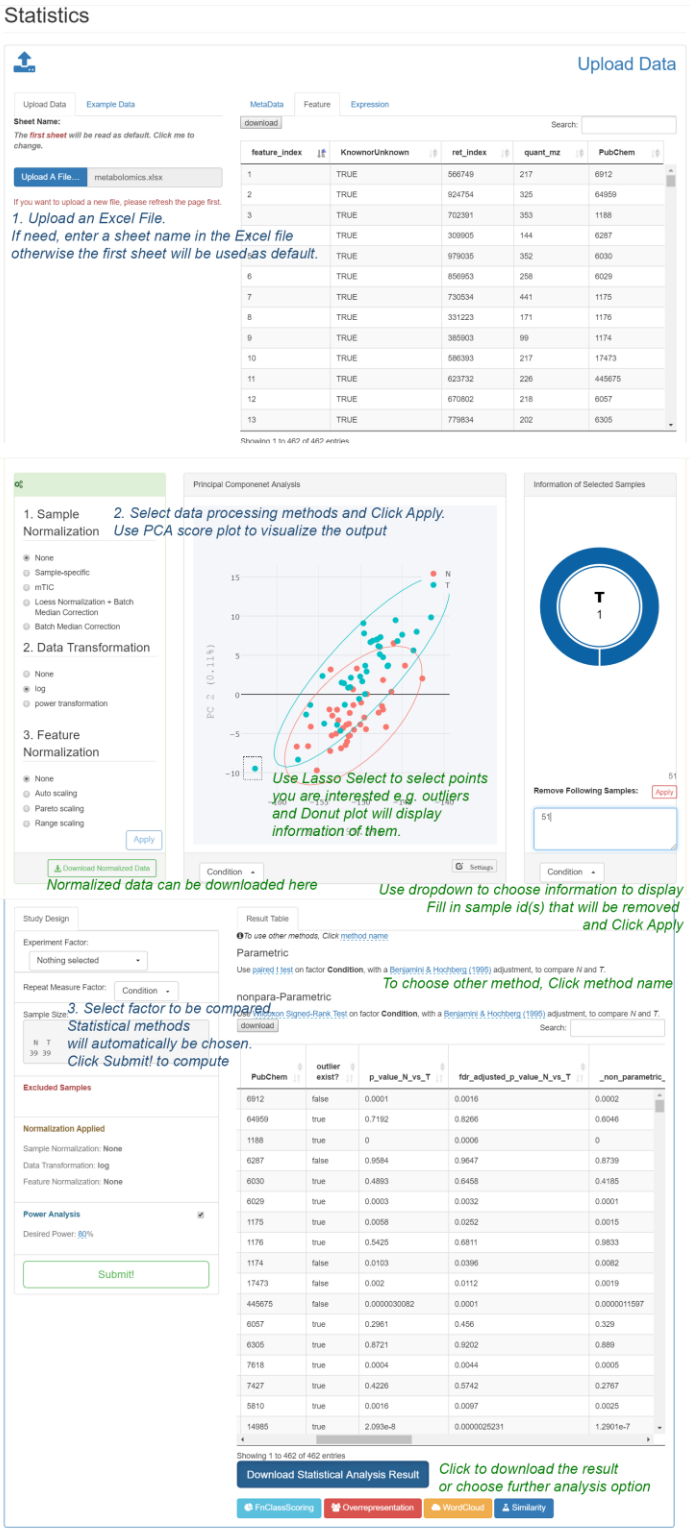

Fig 1. Data normalization and statistical analysis comparing two-paired groups of metabolomic study.

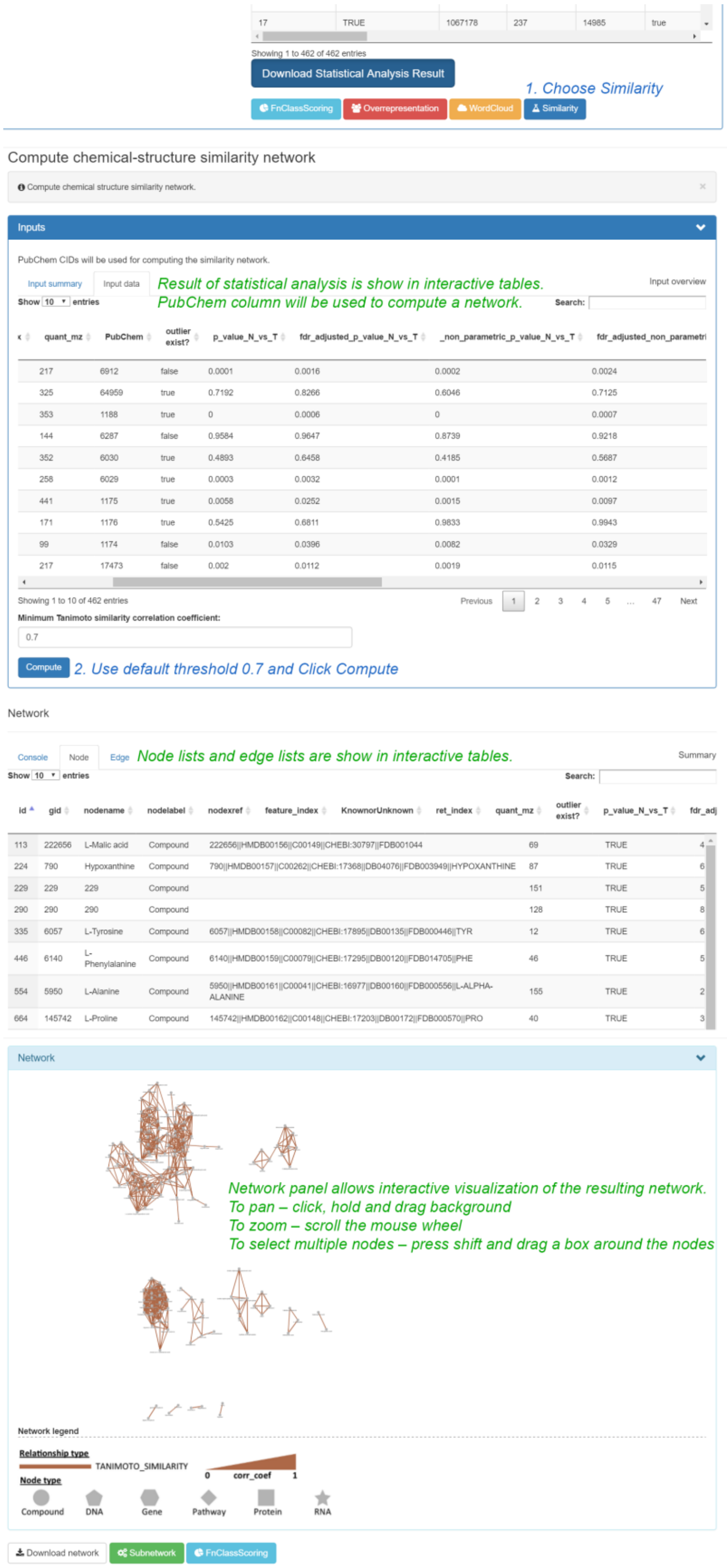

Fig 2. Computing chemical similarity network and performing FnClassScoring on network nodes.

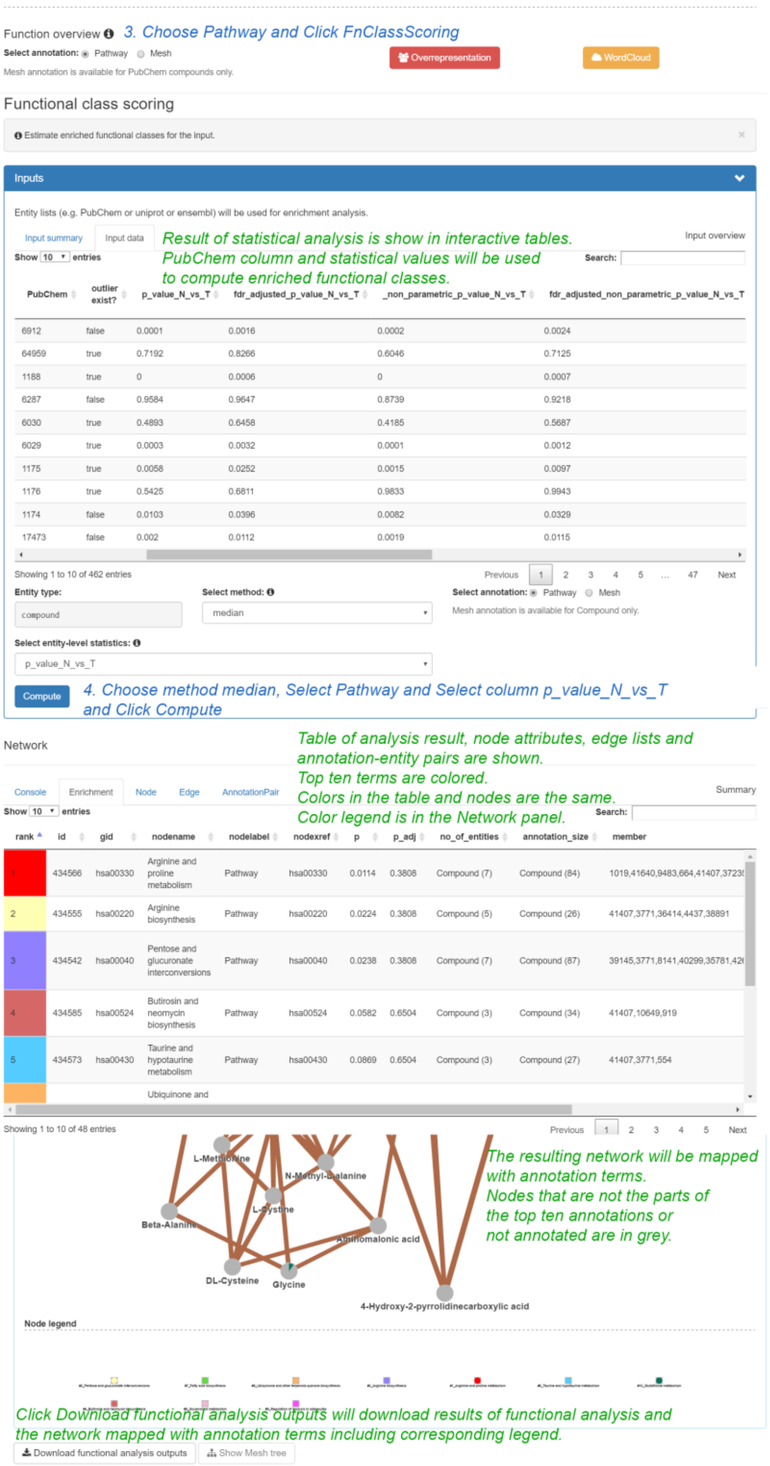
Function overview
3. Choose Pathway and Click FnClassScoring
Select annotation: Pathway Mesh
Mesh annotation is available for PubChem compounds only.
Overrepresentation
WordCloud
Functional class scoring
Estimate enriched functional classes for the input.
Inputs
Entity lists (e.g. PubChem or uniprot or ensembl) will be used for enrichment analysis.
Input summary
Input data
Result of statistical analysis is show in interactive tables.
PubChem column and statistical values will be used
to compute enriched functional classes.
Input overview
Show 10 entries
Search:
PubChem outlier exist? p_value_N_vs_T fdr_adjusted_p_value_N_vs_T _non_parametric_p_value_N_vs_T fdr_adjusted_non_parametric_p_value_N_vs_T
6912 false 0.0001 0.0016 0.0002 0.0024
64959 true 0.7192 0.8266 0.6046 0.7125
1188 true 0 0.0006 0 0.0007
6287 false 0.9584 0.9647 0.8739 0.9218
6030 true 0.4893 0.6458 0.4185 0.5687
6029 true 0.0003 0.0032 0.0001 0.0012
1175 true 0.0058 0.0252 0.0015 0.0097
1176 true 0.5425 0.6811 0.9833 0.9943
1174 false 0.0103 0.0396 0.0082 0.0329
17473 false 0.002 0.0112 0.0019 0.0115
Showing 1 to 10 of 462 entries
Previous 1 2 3 4 5 … 47 Next
Entity type:
compound
Select method:
median
Select annotation: Pathway Mesh
Mesh annotation is available for Compound only.
Select entity-level statistics:
p_value_N_vs_T
Compute
4. Choose method median, Select Pathway and Select column p_value_N_vs_T
and Click Compute
Network
Table of analysis result, node attributes, edge lists and
annotation-entity pairs are shown.
Top ten terms are colored.
Colors in the table and nodes are the same.
Color legend is in the Network panel.
Console
Enrichment
Node
Edge
AnnotationPair
Summary
Show 10 entries
Search:
rank id gid nodename nodelabel nodexref p p_adj no_of_entities annotation_size member
1 434566 hsa00330 Arginine and proline metabolism Pathway hsa00330 0.0114 0.3808 Compound (7) Compound (84) 1019,41640,9483,664,41407,3723
2 434555 hsa00220 Arginine biosynthesis Pathway hsa00220 0.0224 0.3808 Compound (5) Compound (26) 41407,3771,36414,4437,38891
3 434542 hsa00040 Pentose and glucuronate interconversions Pathway hsa00040 0.0238 0.3808 Compound (7) Compound (87) 39145,3771,8141,40299,35781,42
4 434585 hsa00524 Butirosin and neomycin biosynthesis Pathway hsa00524 0.0582 0.6504 Compound (3) Compound (34) 41407,10649,919
5 434573 hsa00430 Taurine and hypotaurine metabolism Pathway hsa00430 0.0869 0.6504 Compound (3) Compound (27) 41407,3771,554
Ubiquinone and
Showing 1 to 10 of 48 entries
Previous 1 2 3 4 5 Next
L-Methionine
N-Methyl-L-alanine
L-Cystine
Beta-Alanine
Aminomalonic acid
DL-Cysteine
Glycine
4-Hydroxy-2-pyrrolidinecarboxylic acid
The resulting network will be mapped
with annotation terms.
Nodes that are not the parts of
the top ten annotations or
not annotated are in grey.
Node legend
Click Download functional analysis outputs will download results of functional analysis and
the network mapped with annotation terms including corresponding legend.
Download functional analysis outputs
Show Mesh tree

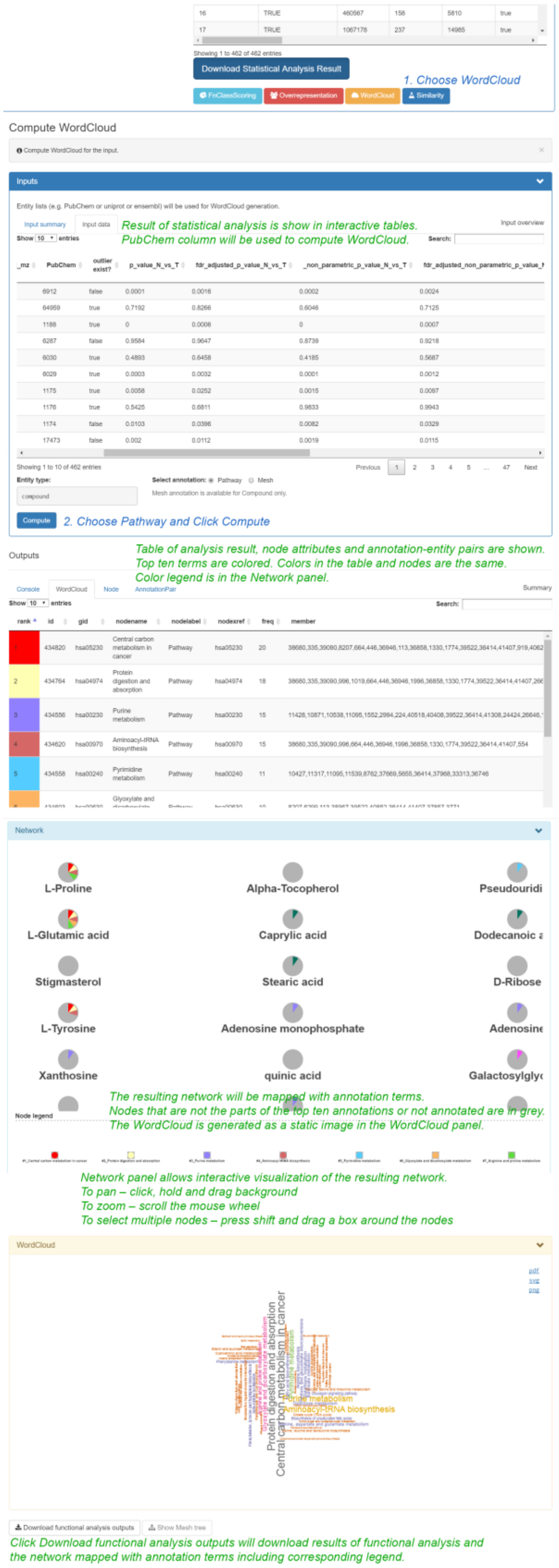

Fig 3. Computing WordCloud for measured compounds.

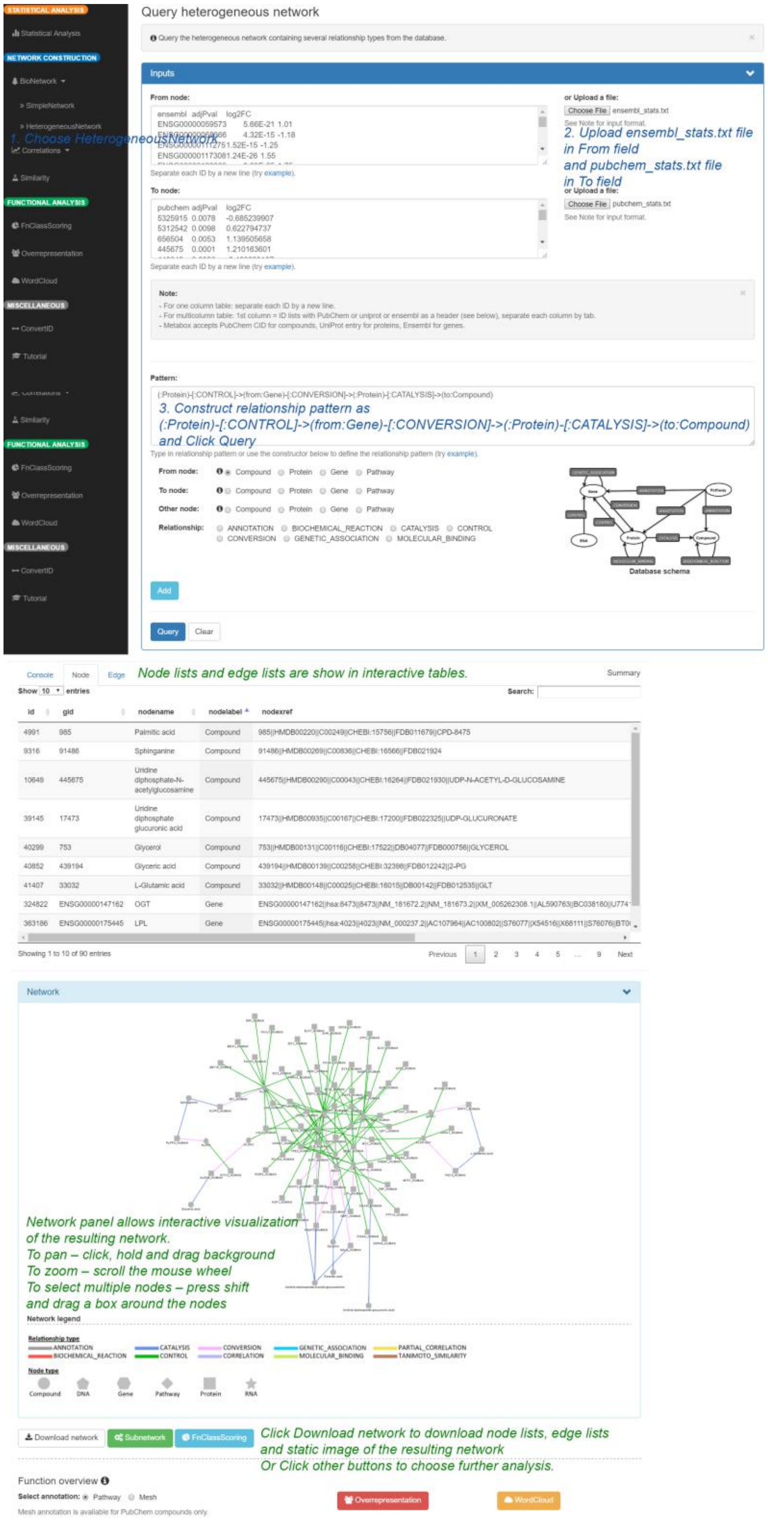

Fig 4. Constructing biological network from gene lists and compound lists. Relationship pattern between genes and compounds is:

(:Protein)-[:CONTROL]->(from:Gene)-[:CONVERSION]->(:Protein)-[:CATALYSIS]->(to:Compound)

REFERENCES

1. Selamat SA, Chung BS, Girard L, Zhang W, Zhang Y, Campan M, et al. Genome-scale analysis of DNA methylation in lung adenocarcinoma and integration with mRNA expression. Genome research. 2012;22(7):1197-211.

2. Wikoff WR, Grapov D, Fahrmann JF, DeFelice B, Rom WN, Pass HI, et al. Metabolomic markers of altered nucleotide metabolism in early stage adenocarcinoma. Cancer prevention research. 2015;8(5):410-8.

3. Fiehn O, Wohlgemuth G, Scholz M. Setup and annotation of metabolomic experiments by integrating biological and mass spectrometric metadata. Lect Notes Comput Sc. 2005;3615:224-39.

4. Barrett T, Wilhite SE, Ledoux P, Evangelista C, Kim IF, Tomashevsky M, et al. NCBI GEO: archive for functional genomics data sets--update. Nucleic acids research. 2013;41(Database issue):D991-5.

5. Shannon P, Markiel A, Ozier O, Baliga NS, Wang JT, Ramage D, et al. Cytoscape: a software environment for integrated models of biomolecular interaction networks. Genome research. 2003;13(11):2498-504.
