# Supplementary material for: Metabox: A Toolbox for Metabolomic Data Analysis, Interpretation and Integrative Exploration
Source: PLoS One. 2017 Jan 31;12(1):e0171046. doi: 10.1371/journal.pone.0171046 (PMC5283729; doi:10.1371/journal.pone.0171046)
Supplement: S3 File — (PDF) [file pone.0171046.s006.pdf]

metabox: a toolbox for metabolomic data analysis, interpretation and integrative exploration

Kwanjeera Wanichthanarak, Sili Fan, Dmitry Grapov, Dinesh Barupal and Oliver Fiehn

9 January 2017

Contents

1 INSTALLATION
1.1 INSTALL metabox
1.2 INSTALL OpenCPU
1.3 INSTALL Neo4j database *(optional)*
2 GRAPHICAL USER INTERFACE
3 WORKFLOWS
3.1 STATISTICS WORKFLOW
3.2 INTERPRETATION WORKFLOW
4 FUNCTIONS
4.1 DATA PROCESSING AND STATISTICAL ANALYSIS
4.2 NETWORK CONSTRUCTION
4.3 FUNCTIONAL ANALYSIS
4.4 CONVERT ID
5 VISUALIZATION
5.1 INTERACTIVE TABLE
5.2 MESH TREE
5.3 WORDCLOUD FIGURE
5.4 INTERACTIVE NETWORK
5.5 FUNCTIONAL ANALYSIS RESULTS
6 REFERENCES

1 INSTALLATION

Metabox can run as a web application locally with OpenCPU single-user server. Follow the steps below to install and run required software packages.

1.1 INSTALL metabox

1) Require R software 3.1.1 or higher (https://www.r-project.org/)
2) Install R package metabox from GitHub by using the following commands in R terminal:

```
## Install R package devtools, if not exist
> install.packages("devtools")

## Install required packages if not exist
> source('https://bioconductor.org/biocLite.R')
> biocLite(c('impute','preprocessCore','GO.db','AnnotationDbi','WGCNA','piano'
,'qpgraph','BioNet','ChemmineR'))

## Install R package metabox
> devtools::install_github("kwanjeeraw/metabox")
> library(metabox)
```

1.2 INSTALL OpenCPU

1) Install OpenCPU single-user server and run the application in a browser by using the following commands in R terminal:

```
## Install OpenCPU single-user server
> install.packages("opencpu")
> library(opencpu)

## Run metabox on a web browser
> opencpu$browse("library/metabox/www")
```

1.3 INSTALL Neo4j database *(optional)*

The Neo4j database is a graph database, a part of the tool for biological network queries and pathway enrichment analysis. Currently the precompiled databases are available on our server for human and will be connected automatically after installing metabox.

The following steps are for setting up a Neo4j database on a local machine.

1) Require Neo4j 3.0.0 or higher (http://neo4j.com/download/).
   Follow installation instructions for each operating system at
   http://neo4j.com/docs/developer-manual/current/#_install_neo4j
2) Download the precompiled database, extract and copy to your Neo4j directory
3) Start the Neo4j server
4) For the first installation of Neo4j, it requires users to setup the password. Go to http://localhost:7474/browser/ for setting the password to *grinn* for the first use.
5) Use the following steps to set a database location for R:

a) For Mac and Linux, Create a file database.R in the folder HOME/USER_NAME/SUBFOLDER_NAME/ e.g. "Users/myname/myfolder/database.R"
b) For Windows, Create a file database.R in the folder HOME/USER_NAME/Documents/ e.g. "Users/myname/Documents/database.R"
c) Copy and paste the following line to the database.R
    a. database.location = "URL OF YOUR DATABASE LOCATION"
    b. Neo4j usually locates at http://localhost:7474/db/data/
    c. e.g. database.location = "http://mylocation.com:7474/db/data/"
d) Reload the web browser

2 GRAPHICAL USER INTERFACE

Graphical user interface (GUI) is compatible on a standard web browser e.g. Chrome, Firefox and Safari. The web page is a two-column layout (Fig 1). A side navigation bar contains the list of different functions and the page content to the right is changed automatically according to the selected function.

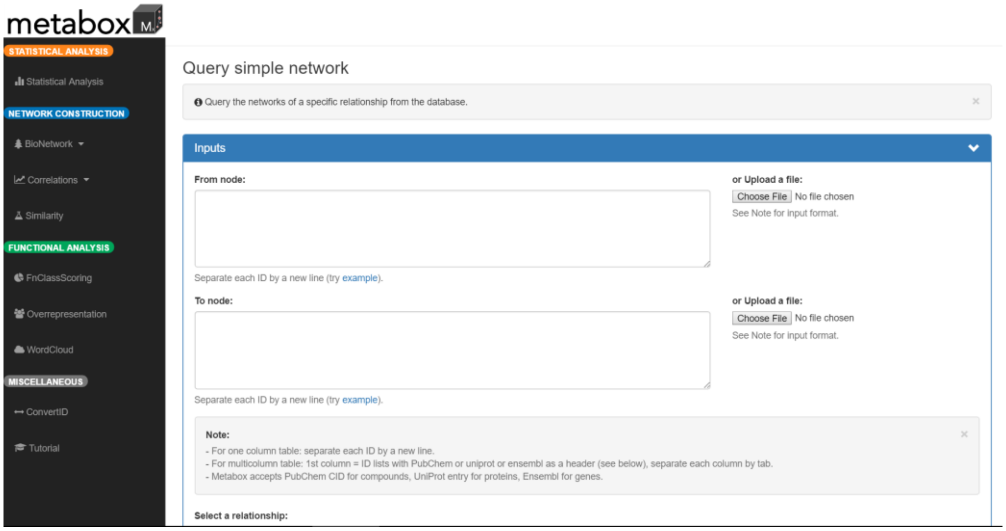

Fig 1. Screenshot of GUI.

3 WORKFLOWS

Metabox supports two different analysis workflows (Fig 2). The tool accepts external inputs and generates outputs at every level of the analysis workflows.

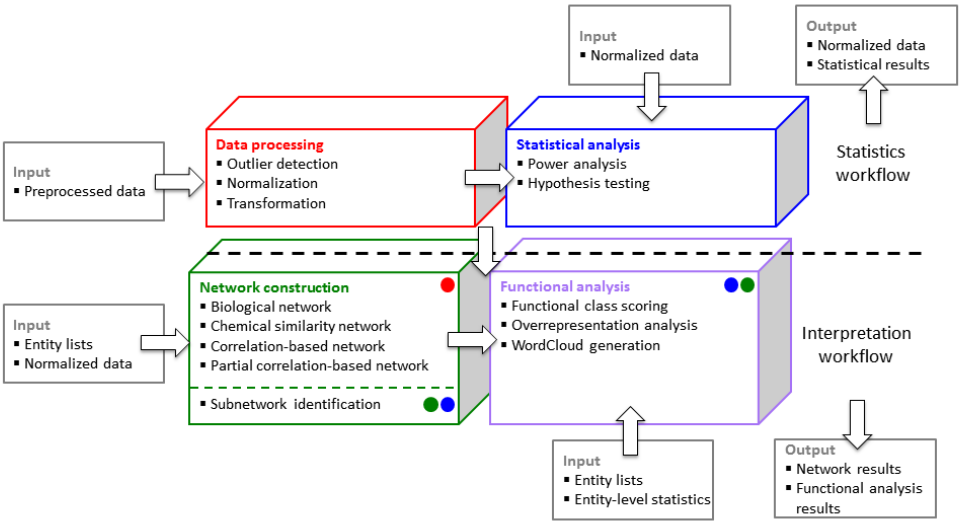

Fig 2. Metabox analysis workflows.

3.1 Statistics workflow

This workflow is for data processing and the identification of significant entities from experimental inputs. It includes the functions for data normalization, data transformation and statistical analysis. The outputs are in a standard file format. The outputs from statistics workflow can also be used directly in the interpretation workflow to be delineated in various contexts including chemical networks, pathway- and chemical-based functions.

Inputs:

- Excel file contains meta-data, features and quantified data (e.g. expression values) using the format in Fig 3 and details are listed in Table 1.

Outputs:

- Comma-separated values (CSV) file with basic statistics, including mean, standard deviation of each experiment group, p-values and adjusted p-values corresponding to experimental design.

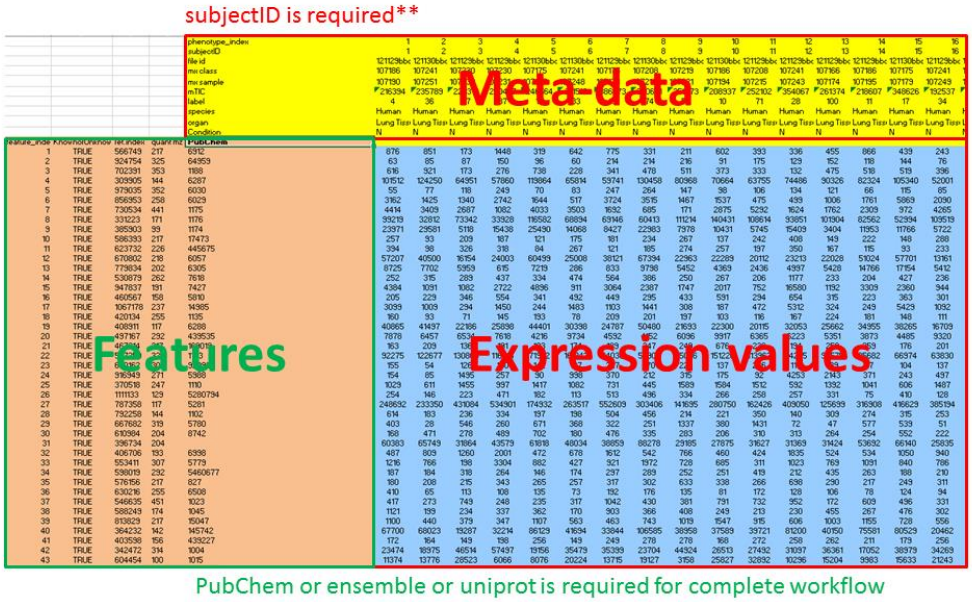

Fig 3. Input format for statistics workflow. Red is a required session. Green is required if you want to use complete workflow. For gene data, header “ensembl” is required for complete workflow. For protein data, “uniprot” is required for complete workflow. For compound data, “PubChem” is require for complete workflow. Detailed information is in Table 1.

Table 1. Summary of headers of input data for statistics workflow.

Name	Description	Example	Required	Note
phenotype_index	Positive Integer. From 1 to the number of total samples.	1,2,3,...,100	NO	If missing, it would be automatically added.
subjectID	Positive integer. From 1 to the number of subjects. Same subject must have same subjectID which will indicate paired-samples.	1,2,3,...,100	YES	If missing, it would be automatically added considering there is no repeated measure.
QC	TRUE or FALSE indicating which sample is quality control. This can be used as calculating RSD and used for loess normalization.	TRUE, FALSE	NO	If missing, there is no QC thus metabox cannot calculate RSD or do loess normalization.
Time_of_Injection	Possitive value. Timestamp. Format can be yyyy-mm-dd HH:MM:SS.	2005-12-24 16:39:58	NO	If missing, cannot do Loess normalization
Batch	Strings indicating batches of samples.	A, B or Batch1, Batch2 etc.	NO	If mising, cannot do Batch Median Correction normalization.
feature_index	Positive Integer. From 1 to the number of total entities.	1,2,3,...,100	NO	If missing, it would be automatically added.
KnownorUnknown	TRUE or FALSE indicating whether it is an known compounds. This is used for mTIC.	TRUE, FALSE	NO	Helpful when doing mTIC normalization
PubChem	PubChem id.	439205	NO	If missing, you can only use statistics workflow but not complete workflow.
ensembl	Ensembl id	ENSG00000166913	NO	If missing, you can only use statistics workflow but not complete workflow.
uniprot	UniProt entry	P31946	NO	If missing, you can only use statistics workflow but not complete workflow.

3.2 Interpretation workflow

The workflow supports the analysis and interpretation of entity lists, processed or normalized data, and entity lists with associated entity-level statistics in biological concepts. It includes the functions to generate different kinds of networks and the options for functional analysis. The workflow accepts both the outputs from the statistics workflow and results from other tools.

Inputs:

- List of entities in a one-column table or multi-column table for Biological network query, Chemical structure similarity, Overrepresentation analysis and WordCloud generation (Fig 4, One-column)
- Tab-delimited text file of multi-column table containing list of entities and associated statistical values for Subnetwork identification and Functional class scoring (Fig 4, Multi-column)
- Tab-delimited text file of quantified data where each row is an entity or variable and columns are samples for Correlation and Partial correlation analysis (Fig 4, Expression table)
- Column header is required for multi-column tables. For gene data, header “ensembl” is required. For protein data, “uniprot” is required. For compound data, “PubChem” is require.

Outputs:

- Tab-delimited text files
- Image files

One-column

ENSG00000175445
ENSG00000123989
ENSG00000239672
ENSG00000115339
ENSG00000140297
ENSG00000198488
ENSG00000068383
ENSG00000143379
ENSG00000241644
ENSG00000123505
ENSG00000117308
ENSG00000109814
ENSG00000105650
ENSG00000205268
ENSG00000113448
ENSG00000160688
ENSG00000173599
ENSG00000134333
ENSG00000002726
ENSG00000068366
ENSG00000237289

Multi-Column

PubChem	adjPval	log2FC
5325915	0.0078	-0.68524
5312542	0.0098	0.622795
656504	0.0053	1.139506
445675	0.0001	1.210164
440043	0.0396	-0.46088
439194	0.0406	0.477637
439176	0.0001	0.866406
145742	0.0076	0.500492
107526	0.001	-1.01618
100714	0.033	0.342076
94270	0.0003	1.095649
94270	0.0003	1.095649
94154	0.009	0.496837
92729	0.0154	0.296059
92092	0.0456	-0.39271
91486	0.0011	-0.62586
65150	0.0011	0.547527
64960	0.0342	-0.25267
33032	0.0058	0.458267
17473	0.0112	0.319933

Expression table

PubChem	T1	T2	T3	T4	T5	T6	T7	T8	T9	T10	N1
439205	8.8009	8.463524	8.280771	8.550747	8.912889	8.430453	8	8.266787	8.330917	7.794416	7.
6287	14.95932	14.50016	14.70315	15.03802	14.86041	14.94059	15.07117	15.04119	15.06251	15.01035	15.
1176	12.01576	11.45943	12.06945	12.18982	11.76777	11.74861	12.30663	12.50009	12.13475	12.41864	12.
1174	11.63436	9.35975	7.761551	8.693487	12.59945	9.705632	8.903882	9.554589	10.12153	8.357552	8.2
445675	14.15062	13.69729	13.58061	14.00158	14.0784	13.921	9.463524	9.851749	9.636625	9.409391	9.5
17473	9.820179	9.400879	8.67948	8.906891	9.071462	8.768184	9.430453	9.377211	9.197217	9.247928	8.9
6057	14.77684	13.88303	14.12121	14.75994	14.56748	14.14235	14.28338	14.34748	14.37198	14.27037	14.
6305	12.15703	11.60825	12.04474	12.15006	12.18797	12.29806	12.05019	12.08281	11.98797	11.90727	12.
5810	9.047124	8.921841	8.693487	8.294621	9.063395	8.511753	8.011227	9.152285	7.857981	7.954196	8.0
6288	15.61646	15.05698	15.07431	15.2862	15.4166	15.17028	15.25577	15.50718	15.15217	15.00453	14.
1123	6.965784	6.066089	6.658211	6.599913	6.491853	6.584963	6.426265	6.409391	6.83289	6.247928	6.1
439312	11.06878	10.38909	10.59712	10.94691	11.04985	10.91364	6.569856	6.087463	6.523562	6.169925	6.3
5988	8.129283	1	5.672425	5.087463	7.70044	6.169925	7.312883	4.523562	5.807355	8.857981	6.3
1110	8.906891	5.807355	8.290019	8.375039	8.60733	8.055282	8.535275	8.936638	8.535275	8.897845	8.2
5281	16.62104	15.83333	16.40939	16.35049	16.56133	15.79238	16.21603	16.47071	16.52182	16.34918	15.
5780	16.29336	16.01676	16.19608	16.22466	16.09358	16.08402	9.211888	9.204571	9.049849	9.103288	9.6
441432	12	9.548822	10.56701	11.35645	11.82377	11.6786	6.84549	6.97728	6.70044	6.491853	6.2
5951	14.21158	13.67121	13.71607	13.94141	14.0921	13.79228	14.57784	14.91439	14.21379	14.4641	14.
6998	10.75822	9.326429	9.394463	10.22641	9.753217	9.990104	9.61471	10.35755	10.86109	11.04371	11.
33037	6.942515	6.672425	6.247928	6.149747	6.584963	6.569856	7.72792	7.584963	6.266787	6.584963	6.1

Fig 4. Input formats for Network construction and Functional analysis.

4 FUNCTIONS

Metabox allows comprehensive analyses of metabolomic data by including several statistical methods to process and identify keys entities of input experiments, and providing different integrative analysis methodologies to facilitate biological interpretation.

4.1 DATA PROCESSING AND STATISTICAL ANALYSIS

4.1.1 Data processing

Data processing procedure includes different kinds of normalization methods and outlier detection (Fig 5).

Normalization method includes sample normalization (mTIC normalization, loess normalization and batch median normalization), data transformation (log and power transformation) and data scaling (auto scaling, pareto scaling and range scaling).

Furthermore, users are able to detect the outlier samples using principal component analysis (PCA) score plot. Then users could further decide on whether to remove them before further analysis or keep them and carry on regardless.

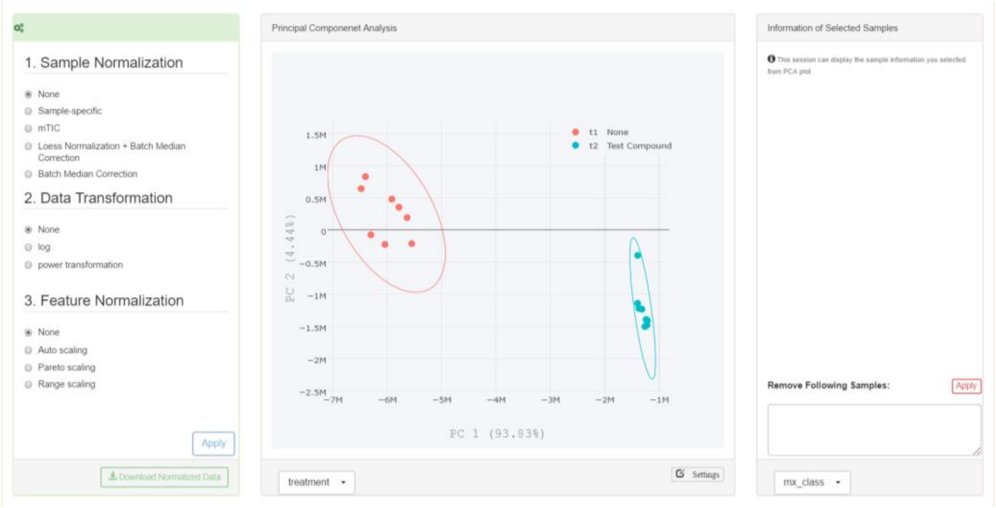

Fig 5. Screenshot of Data Processing panel. Principal component analysis (PCA) score plot is used for real-time visualization during data processing procedures. It allows users to detect outliers and choose appropriate methods for data normalization and transformation. Users are able to select scatters on the PCA score plot, get the corresponding sample information from a donut chart and can remove unwanted samples.

4.2.1 Statistical Analysis

Currently only univariate statistical analysis is available. Different hypothesis testing procedures can be applied to different study designs accordingly. Possible study design types (default hypothesis testing methods) are

- One independent factor with two levels (Welch t test, Mann-Whitney U test)

- One independent factor with multiple levels (Welch one way ANOVA, Kruskal–Wallis one way ANOVA, post hoc analysis: Games Howell test, Dunn's test with Bonferroni adjustment)
- One repeated-measure factor with two levels (paired t test,Wilcoxon signed-rank test)
- One repeated-measure factor with multiple levels (one way repeated ANOVA with Greenhouse-Geisser adjustment, Friedman test, post hoc: paired t test with Bonferroni adjustment, Wilcoxon signed-rank test with a Bonferroni adjustment)
- Two independent factors (two way ANOVA, two way ANOVA with robust estimation)
- Two repeated-measure factors (two way repeated ANOVA)
- Mixed factors with one independent factor and one repeated-measure factor (mixed ANOVA)

For simple study design (one factor cases), Benjamini–Hochberg procedure (or post hoc procedure for multi-levels cases) will be performed to deal with multiple comparison problems. For complex study design (two-factor cases), a thorough analysis on all the possible combination of levels (with post hoc procedure) would be performed. This means that after testing for interaction between two factors, main effect and simple main effect will also be tested, followed by corresponding post hoc analysis.

Except two repeated-measure factors case and mixed factor case, non-parametric tests are provided as default to eliminate the effects of violation of the parametric test assumptions.

Inputting the study design type is simple (Fig 6, left). User could just select the factor name listed in Experiment Factor within Study Design panel and metabox will automatically choose the above listed hypothesis testing after required processing procedure. In addition, users can also select hypothesis testing other than default settings (Fig 6, top).

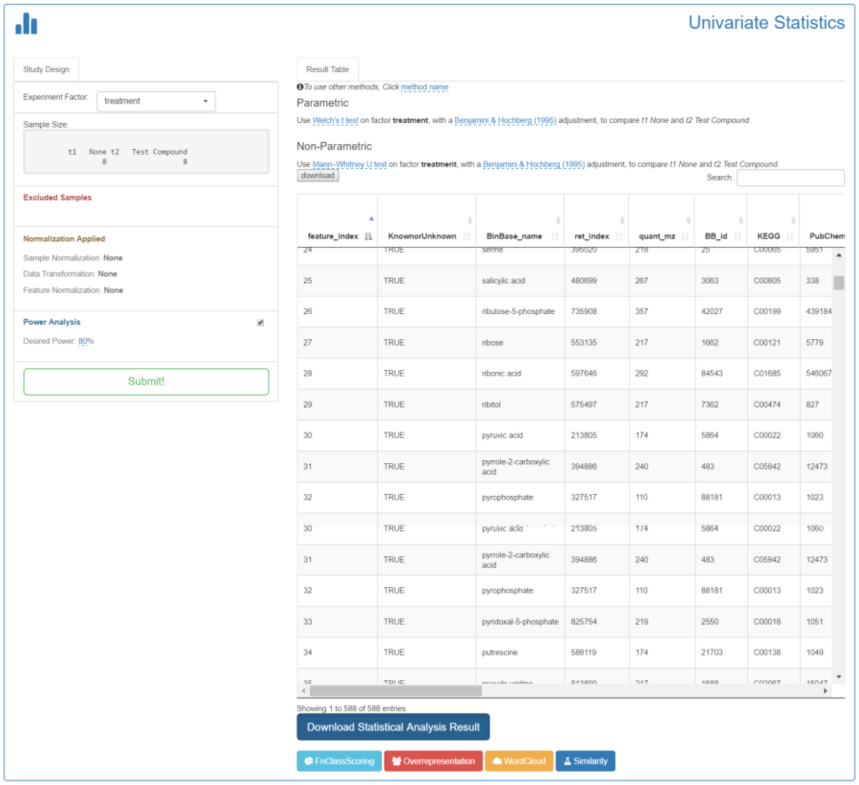

Fig 6. Screenshot of Univariate Statistics panel and resulting table from the analysis. Study Design panel is for selection of experimental factor and power value for the statistical analysis (left). Metabox will automatically choose hypothesis testing methods, however, users can also select other methods if need (top). The result can be downloaded or transferred to other analysis modules.

4.2 NETWORK CONSTRUCTION

Several approaches are included to generate networks in different contexts.

4.2.1 Biological network query (BioNetwork)

The function supports the integrative exploration of biological entities in the context of biological networks. The Neo4j graph database is required here. The database contains domain knowledge relationships among a variety of biological entities such as gene-encode-protein associations, protein-compound catalytic reactions and substrate-product pairs (Fig 7).

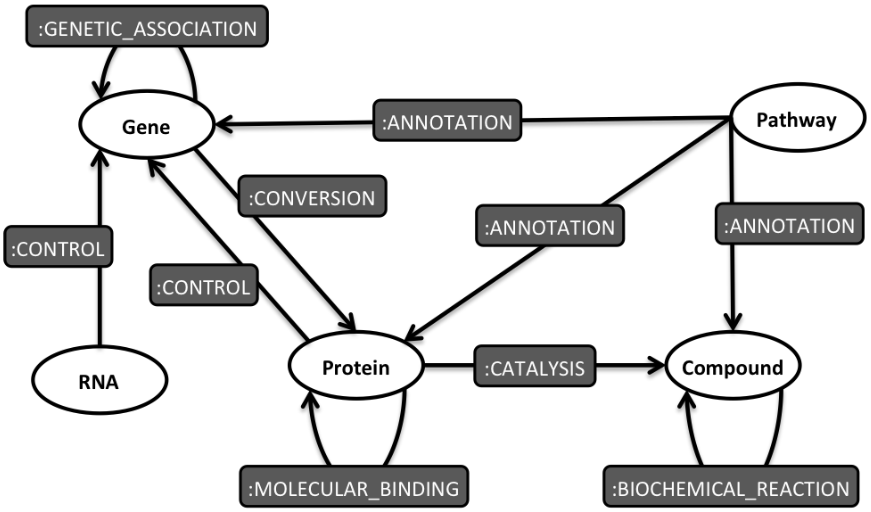

Fig 7. Database schema. Ovals denote biological entities and round rectangular indicate relationships.

There are two options to query biological networks from the database: SimpleNetwork and HeterogeneousNetwork. SimpleNetwork is to query biological networks of a specific relationship. Here we provide a list of relationships where users can choose to query (Fig 8).

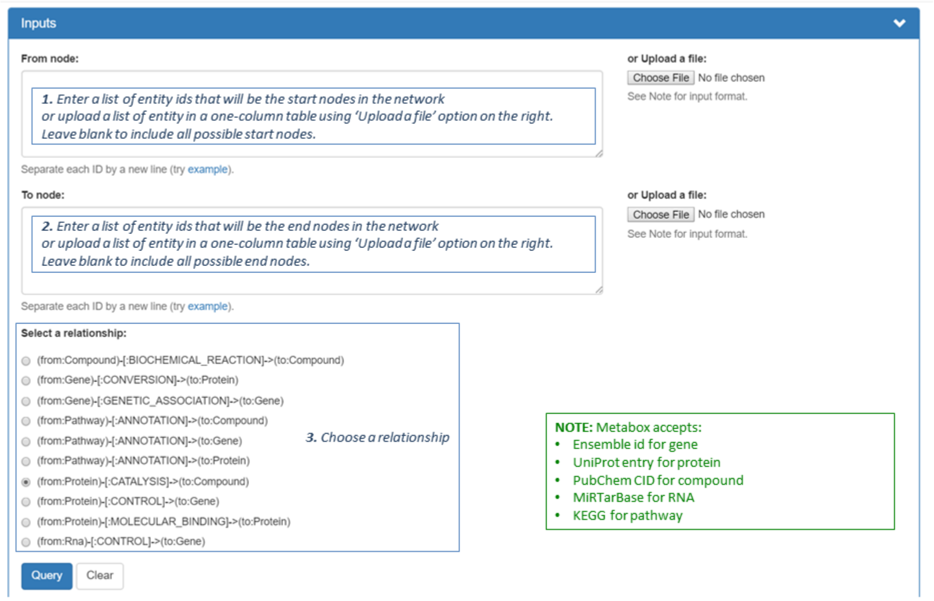

Fig 8. SimpleNetwork option. Steps to query biological networks are listed in blue boxes and a green box contains related explanation.

HeterogeneousNetwork is to query biological networks containing one or several relationship types. Here users can use the constructor to provide relationship pattern for the query (Fig 9).

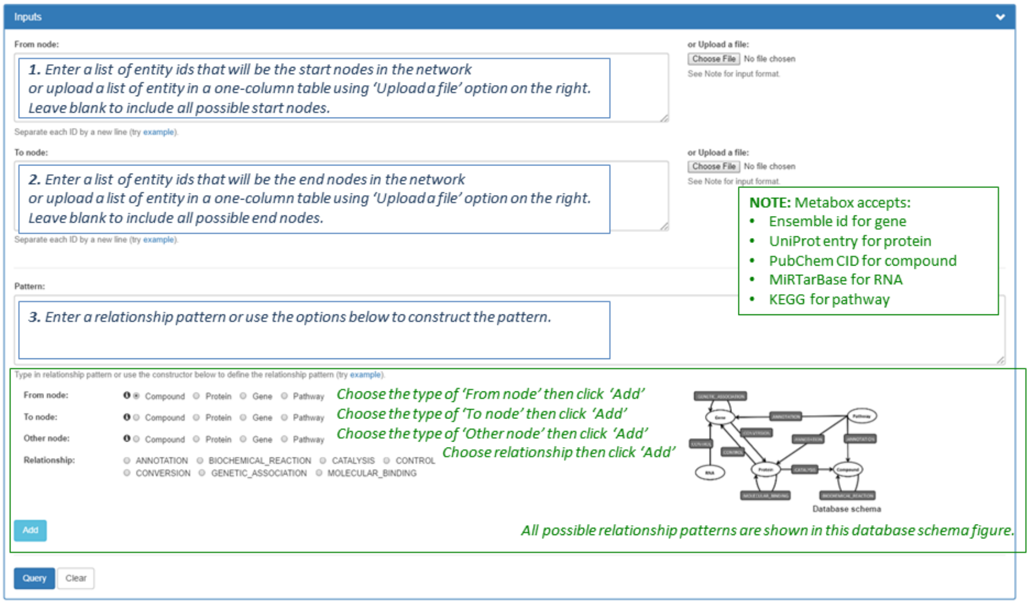

Fig 9. HeterogeneousNetwork option. Steps to query biological networks are listed in blue boxes and a green box includes explanation.

The queried network can be visualized interactively. Node and edge lists are provided and can be downloaded as tab-delimited text files. The network can be analyzed further in the scope of subnetwork, functional class scoring (or set enrichment analysis), overrepresentation analysis and WordCloud generation (Fig 10).

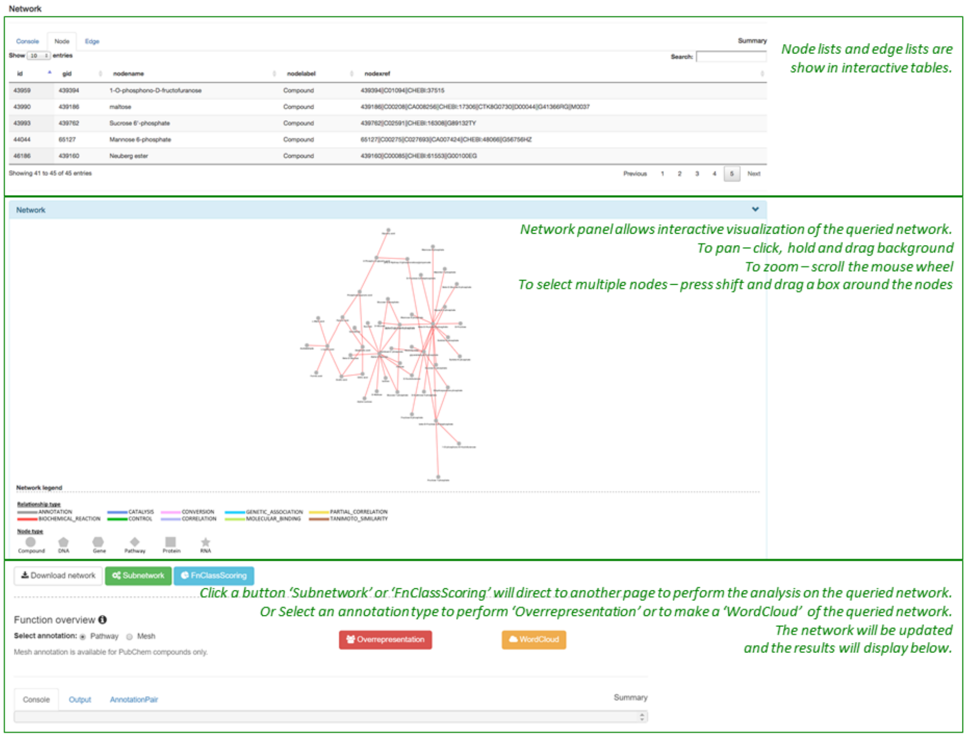

Fig 10. Network visualization and functional analysis.

4.2.2 Correlations

We include both pairwise and partial correlation analysis approaches to estimate empirical relationships from quantified data (see Fig 11 for Inputs panel). The pairwise correlation, including Pearson, Spearman or Kendall correlation is based on WGCNA R package (1), and the partial correlation is based on qpgraph R package (2, 3). Similar to BioNetwork, the correlation networks can be visualized interactively. Node and edge lists are shown in interactive tables and can be downloaded as tab-delimited text files. The network can be analyzed further in the scope of subnetwork, functional class scoring (or set enrichment analysis), overrepresentation analysis and WordCloud generation.

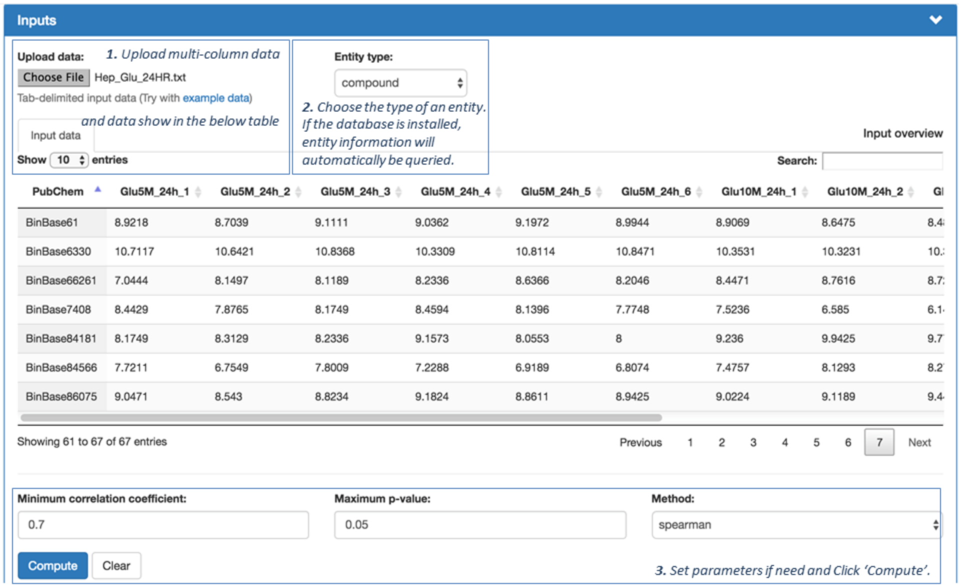

Fig 11. Inputs panel of Correlations function. Steps to compute correlation networks are listed in blue boxes.

4.2.3 Similarity

The function computes a chemical structure similarity network for the list of PubChem compounds (PubChem CIDs) (Fig 12). The chemical-based network is computed from PubChem substructure fingerprints using chemical similarity searching approach (4, 5). Similar to BioNetwork and Correlations, the similarity networks can be visualized interactively. Node and edge lists are shown in interactive tables and can be downloaded as tab-delimited text files. The network can be analyzed further in the scope of subnetwork, functional class scoring (or set enrichment analysis), overrepresentation analysis and WordCloud generation.

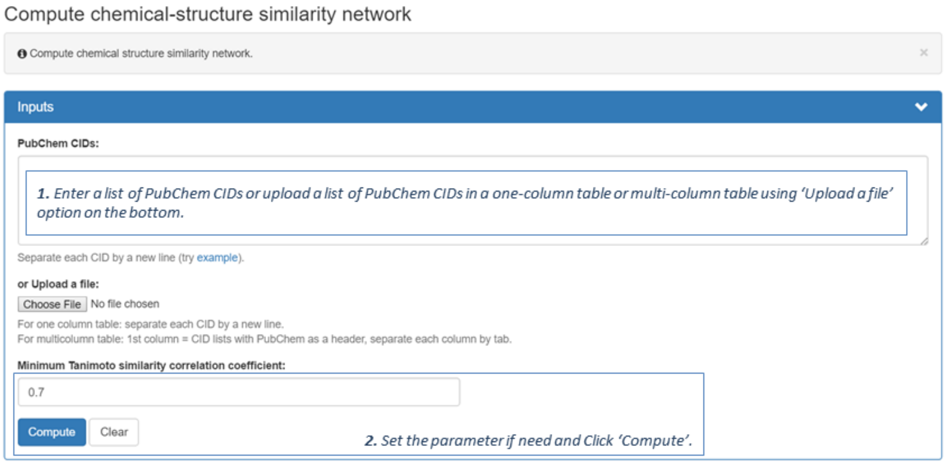

Fig 12. Inputs panel of Similarity function. Steps to compute similarity networks are listed in blue boxes.

4.2.4 Subnetwork

The function identifies an active subnetwork of an input network generated by BioNetwork, Correlations and Similarity using entity-level statistics (Fig 13). This approach is based on BioNet R package (6, 7), which identifies the subnetwork by computing node scores and using a heuristic search for the high-scoring subnetwork. Similar to BioNetwork, Correlations and Similarity, the subnetworks can be visualized interactively. Node and edge lists are shown in interactive tables and can be downloaded as tab-delimited text files. The network can be analyzed further in the scope of functional class scoring (or set enrichment analysis), overrepresentation analysis and WordCloud generation.

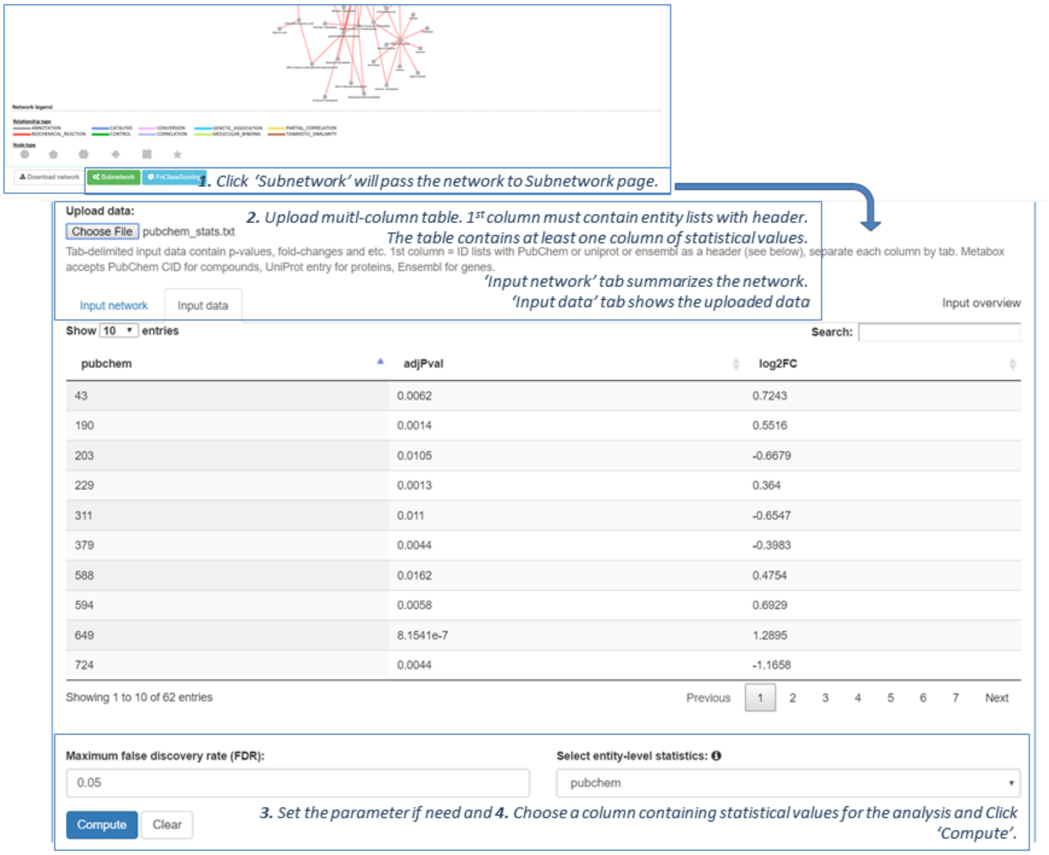

Fig 13. Subnetwork option. Steps to compute a subnetwork are listed in blue boxes.

4.3 FUNCTIONAL ANALYSIS

Three different approaches are provided to support functional analysis of entity lists or network nodes. Functional interpretations in the context of KEGG pathways are available for all entity types and the analysis in the scope of Medical Subject Headings (MeSH) (8) chemicals and drugs category from PubChem is included for compounds.

4.3.1 Functional class scoring

Functional class scoring or set enrichment analysis evaluates the significance of annotation terms using entity-level statistics. The function is based on an R package Piano (9) that contains widely used methods for this analysis including Reporter features (10, 11), Fisher's method (12), Stouffer's method (13), Median and Mean. Metabox allows the analysis for network nodes (Fig 14A) as a result of BioNetwork, Correlations, Similarity and Subnetwork and for input entities (Fig 14B).

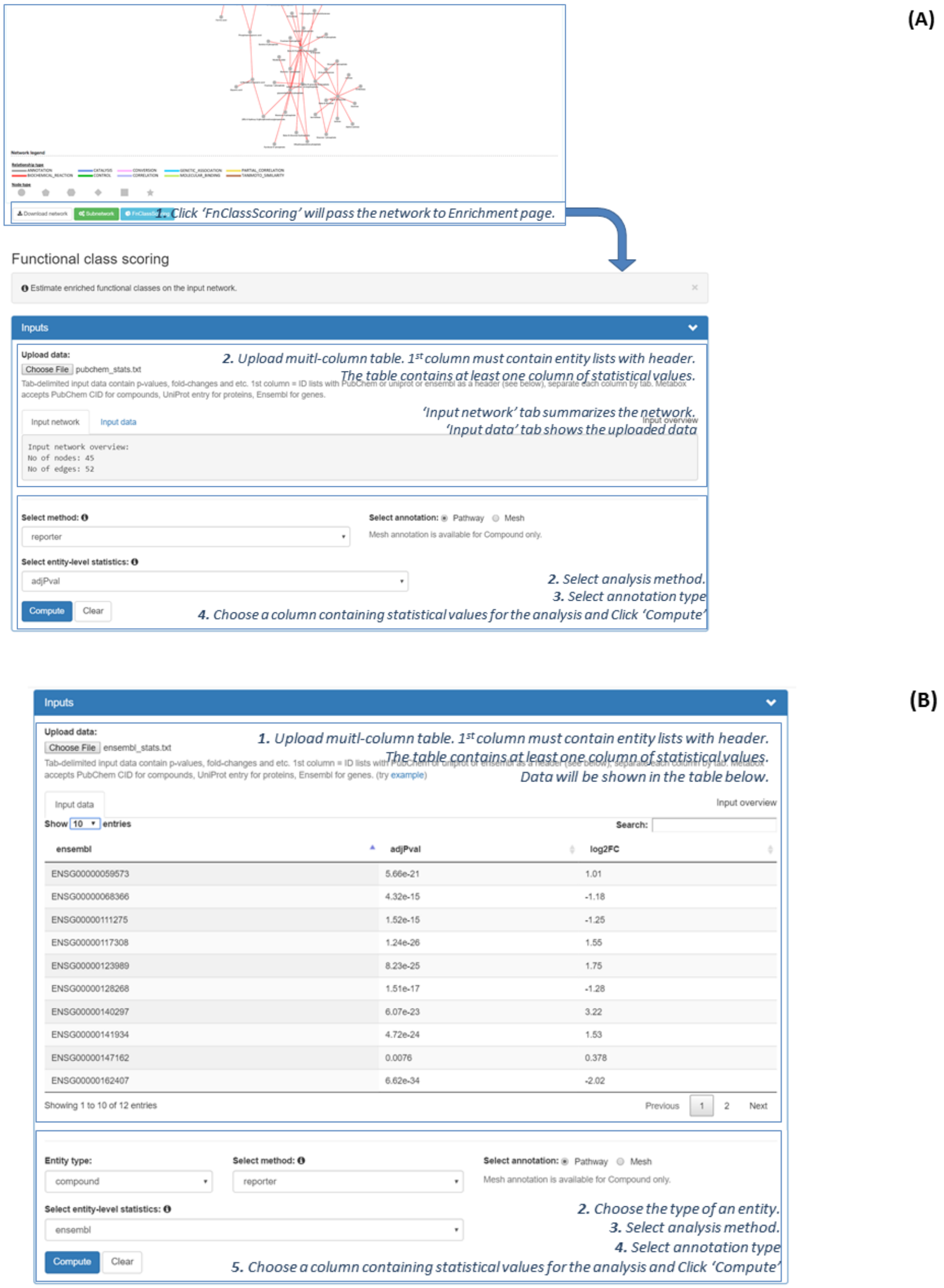

Fig 14. FnClassScoring option. Functional class scoring for the input network (A) for the input entities (B). Steps are listed in blue boxes.

4.3.2 Overrepresentation

The function is to identify overrepresented functional terms for the given list of preselected entities using hypergeometric test. Similar to Enrichment, the overrepresentation analysis can be performed on network nodes (Fig 15A) as a result of BioNetwork, Correlations, Similarity and Subnetwork and for input entities (Fig 15B).

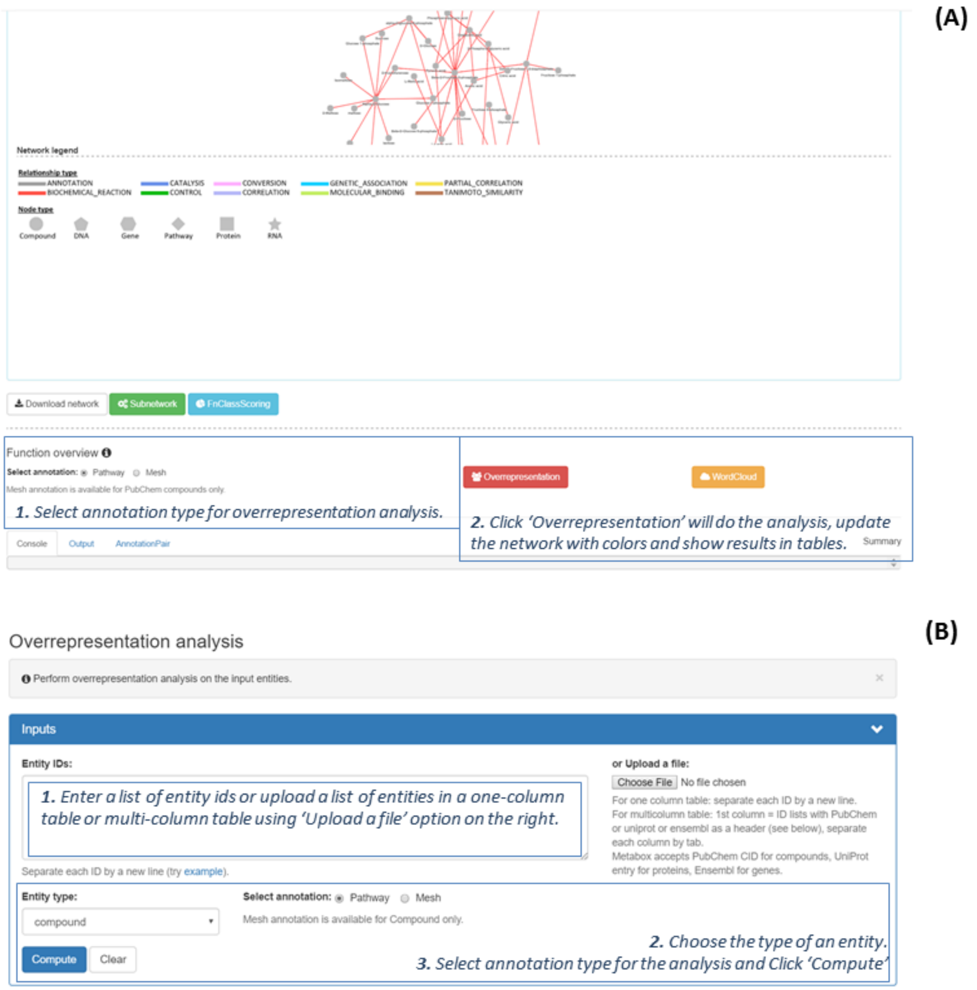

Fig 15. Overrepresentation option. Overrepresentation analysis for the network nodes (A) and overrepresentation analysis for the input entities (B). Steps are listed in blue boxes.

4.3.3 WordCloud

A word cloud is a simple, graphical presentation of words in which the size of a word corresponding to its frequency. It provides a quick summary of annotation terms of the given entities. Similar to Enrichment and Overrepresentation, the WordCloud generation can be performed on network nodes (Fig 16A) as a result of BioNetwork, Correlations, Similarity and Subnetwork and for input entities (Fig 16B).

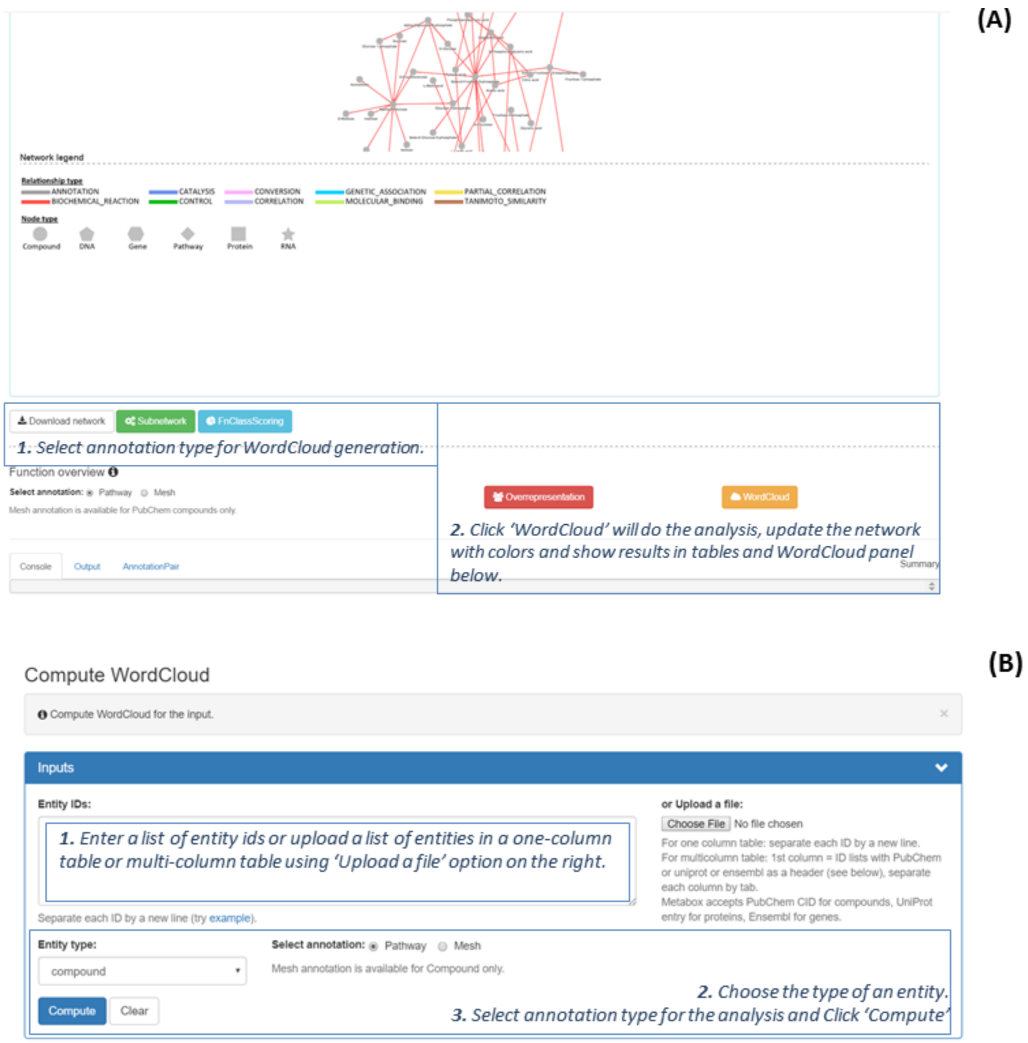

Fig 16. WordCloud option. WordCloud generation for the network nodes (A) and WordCloud generation for the input entities (B). Steps are listed in blue boxes.

4.4 CONVERT ID

We include an option to convert input entities to Neo4j internal ids (NIDs) and Grinn ids (GIDs) (Fig 17). The function accepts name of entities or cross-reference ids e.g. KEGG ids.

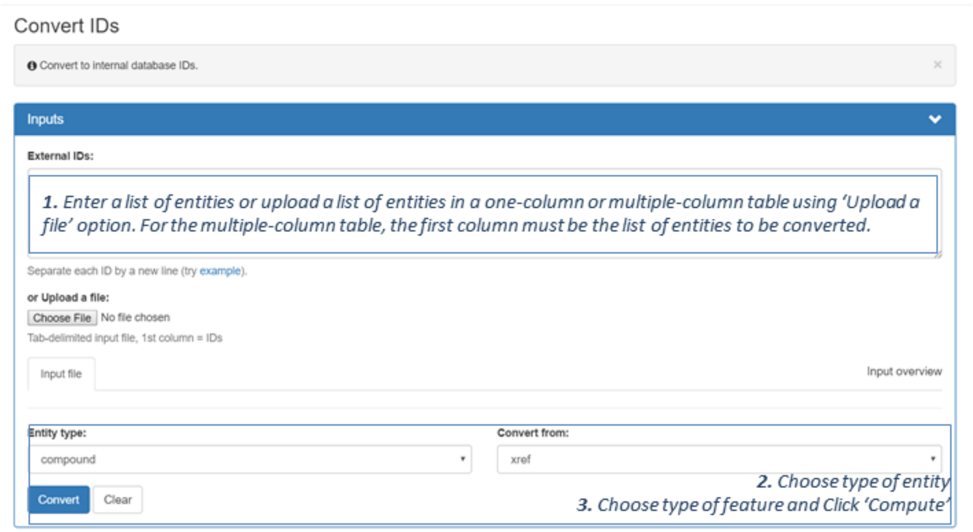

Fig 17. ConvertID option. Steps are listed in blue boxes.

5 VISUALIZATION

5.1 Interactive table

The interactive table is used to display multi-column input data and results. Users can customize the number of entries to show, sort data by a specific column and search data in the table (Fig 18). In addition, table outputs of functional analysis will be colored to illustrate top ten annotation terms (See section 5.5 Functional analysis results for details).

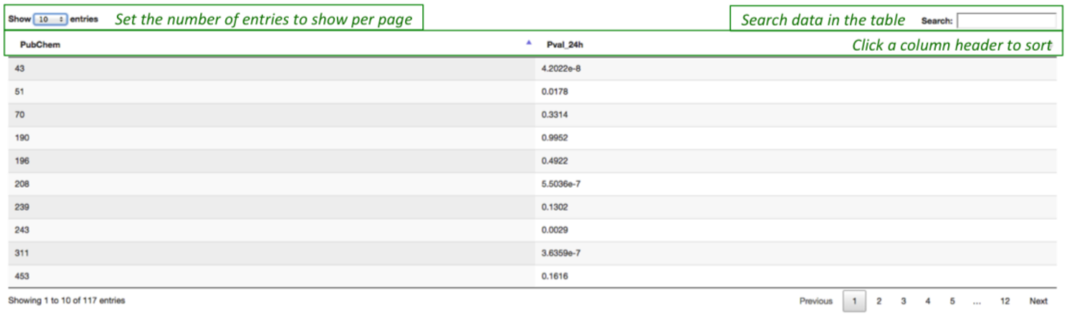

PubChem	Pval_24h
43	4.2022e-8
51	0.0178
70	0.3314
190	0.9952
196	0.4922
208	5.5036e-7
239	0.1302
243	0.0029
311	3.6359e-7
453	0.1616

Fig 18. Interactive table.

5.2 MeSH tree

The results for MeSH annotations are displayed as a tree in which color scale is ranging from yellow to red (Fig 19). Yellow denotes high p-values for FnClassScoring and Overrepresentation, or small number of frequency for WordCloud, whereas red scale denotes low p-values for FnClassScoring and Overrepresentation, or large number of frequency for WordCloud.

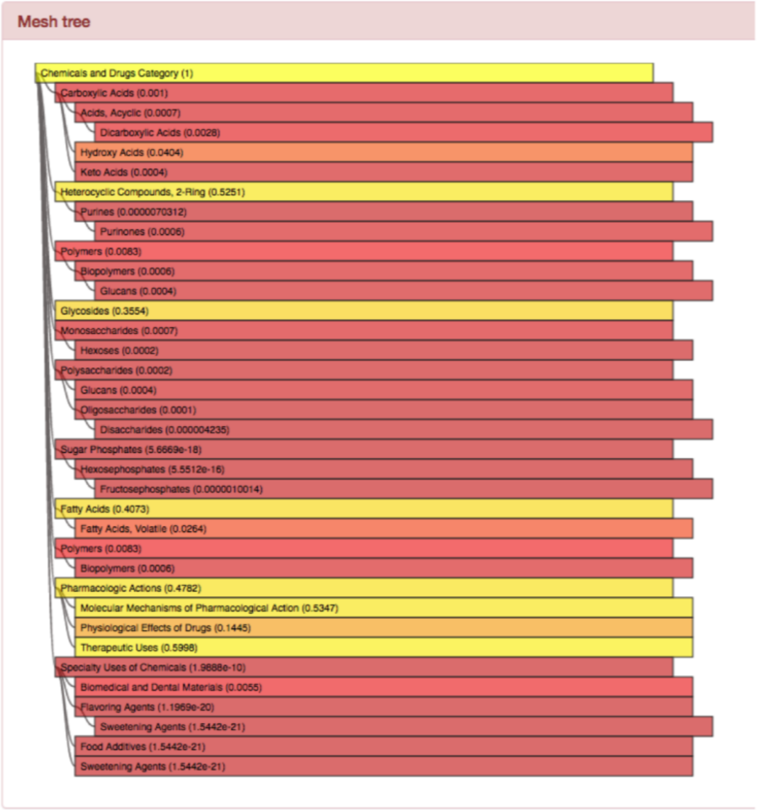

Fig 19. MeSH tree.

5.3 WordCloud figure

WordCloud panel shows a static image of WordCloud in which the font size and color corresponding to word frequency (i.e. the number of input entities in an annotation term) (Fig 20). The image can be downloaded in different file formats such as PDF, PNG and SVG.

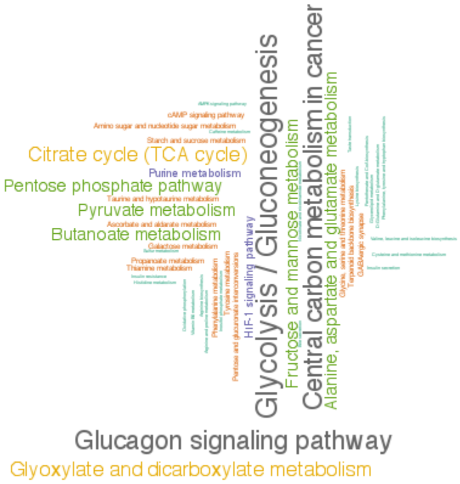

Fig 20. WordCloud.

5.4 Interactive network

Network outputs can be interactively explored in the Network panel. Using a mouse or a touchpad can do network navigation such as pan, zoom and select. Network legend is included at the bottom of the panel. Thickness of edges conforms correlation coefficient for weighted-correlation and similarity networks. Solid and dashed lines denote positive and negative correlations respectively. The network will be updated and network nodes will be colored after functional analysis (See section 5.5 Functional analysis results for details).

5.5 Functional analysis results

The results of functional analysis functions including FnClassScoring, Overrepresentation and WordCloud are presented in a table (Fig 21) and a network form (Fig 22).

Enrichment table, Overrepresentation table and WordCloud table contain a rank column, which is sorted by p-values for FnClassScoring and Overrepresentation, or by frequency for WordCloud. Top ten annotation terms will be colored and the color legend is illustrated in the Network panel. The tables include statistical values of annotation terms, number of input entities and the list of entities of each annotation term.

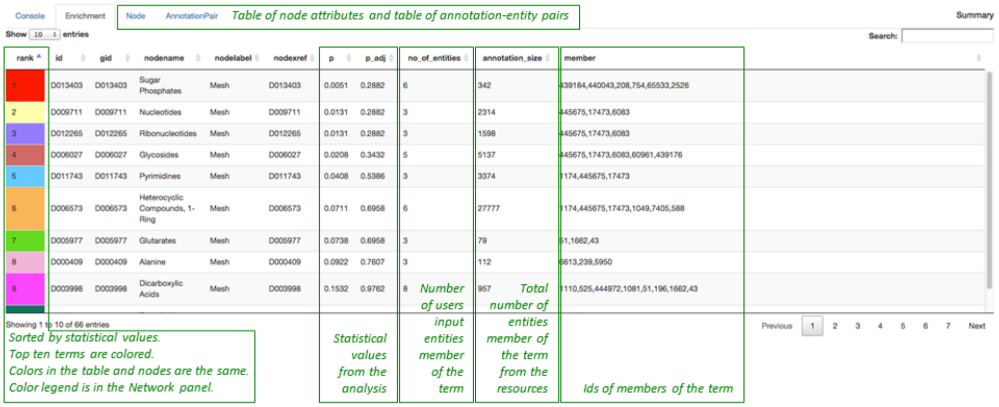

Fig 21. Table form of functional analysis result. The outputs of functional analysis (FnClassScoring in this figure) compose of three tables. The Enrichment table is shown here.

Network nodes or input entities in the Network panel are shown with a pie-chart format in which colors represent top ten annotation terms from the analysis. The color legend is automatically generated in the Network panel and colors also show in the result tables. Pie size does not reflect any typical value. Each node can totally contain ten pies if it belongs to the top ten annotation terms. Nodes that are not the parts of the top ten annotations or not annotated are in grey.

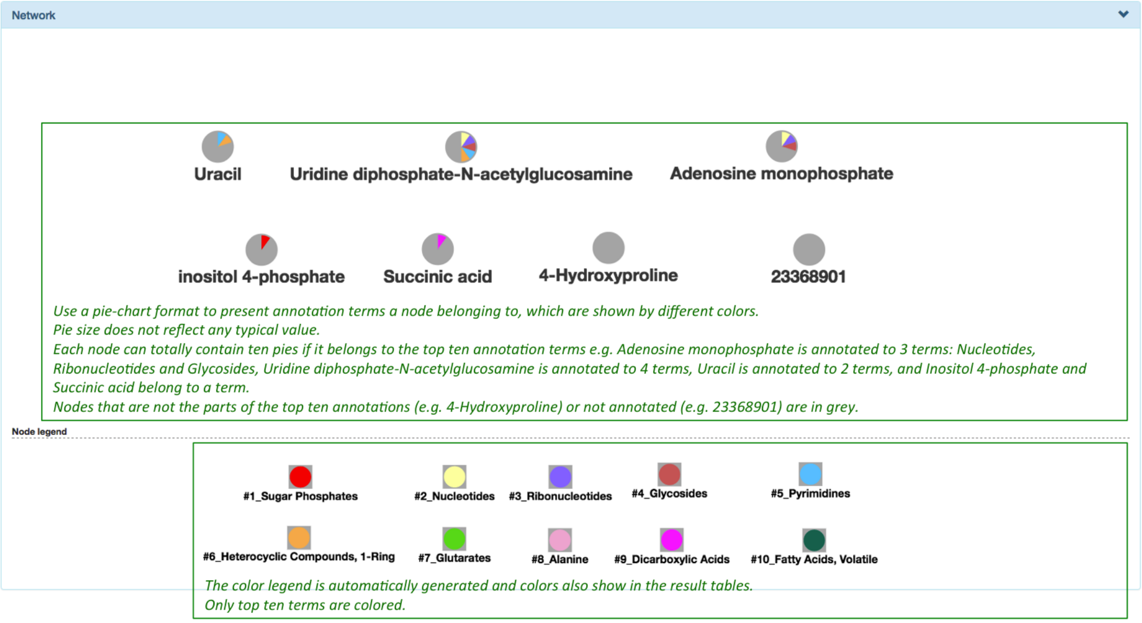

Fig 22. Network form of functional analysis result. The network output of functional analysis (FnClassScoring in this figure) is shown with a pie-chart format.

6 REFERENCES

1. Langfelder P, Horvath S. WGCNA: an R package for weighted correlation network analysis. BMC bioinformatics. 2008;9:559.

2. Tur I, Roverato A, Castelo R. Mapping eQTL networks with mixed graphical Markov models. Genetics. 2014;198(4):1377-93.

3. Castelo R, Roverato A. Reverse engineering molecular regulatory networks from microarray data with qp-graphs. Journal of computational biology : a journal of computational molecular cell biology. 2009;16(2):213-27.

4. Barupal DK, Haldiya PK, Wohlgemuth G, Kind T, Kothari SL, Pinkerton KE, et al. MetaMapp: mapping and visualizing metabolomic data by integrating information from biochemical pathways and chemical and mass spectral similarity. BMC bioinformatics. 2012;13:99.

5. Willett P. Chemical Similarity Searching. Journal of Chemical Information and Modeling. 1998;38(6):983-96.

6. Beisser D, Klau GW, Dandekar T, Muller T, Dittrich MT. BioNet: an R-Package for the functional analysis of biological networks. Bioinformatics. 2010;26(8):1129-30.

7. Dittrich MT, Klau GW, Rosenwald A, Dandekar T, Muller T. Identifying functional modules in protein-protein interaction networks: an integrated exact approach. Bioinformatics. 2008;24(13):i223-31.

8. Lipscomb CE. Medical Subject Headings (MeSH). Bulletin of the Medical Library Association. 2000;88(3):265-6.

9. Varemo L, Nielsen J, Nookaew I. Enriching the gene set analysis of genome-wide data by incorporating directionality of gene expression and combining statistical hypotheses and methods. Nucleic acids research. 2013;41(8):4378-91.

10. Oliveira A, Patil K, Nielsen J. Architecture of transcriptional regulatory circuits is knitted over the topology of bio-molecular interaction networks. BMC systems biology. 2008;2(1):17.

11. Patil KR, Nielsen J. Uncovering transcriptional regulation of metabolism by using metabolic network topology. Proceedings of the National Academy of Sciences. 2005;102(8):2685-9.

12. Fisher RA. Statistical methods for research workers. Edinburgh, London,: Oliver and Boyd; 1925. ix p., 1 l., p.

13. Stouffer SA. The American soldier. Princeton,: Princeton University Press; 1949.
